# Attenuation of a very virulent Marek's disease herpesvirus (MDV) by codon pair bias deoptimization

**DOI:** 10.1371/journal.ppat.1006857

**Published:** 2018-01-29

**Authors:** Kathrin Eschke, Jakob Trimpert, Nikolaus Osterrieder, Dusan Kunec

**Affiliations:** Institut für Virologie, Zentrum für Infektionsmedizin, Freie Universität Berlin, Berlin, Germany; Emory Vaccine Center, UNITED STATES

## Abstract

Codon pair bias deoptimization (CPBD) has enabled highly efficient and rapid attenuation of RNA viruses. The technique relies on recoding of viral genes by increasing the number of codon pairs that are statistically underrepresented in protein coding genes of the viral host without changing the amino acid sequence of the encoded proteins. Utilization of naturally underrepresented codon pairs reduces protein production of recoded genes and directly causes virus attenuation. As a result, the mutant virus is antigenically identical with the parental virus, but virulence is reduced or absent. Our goal was to determine if a virus with a large double-stranded DNA genome, highly oncogenic Marek’s disease virus (MDV), can be attenuated by CPBD. We recoded UL30 that encodes the catalytic subunit of the viral DNA polymerase to minimize (deoptimization), maximize (optimization), or preserve (randomization) the level of overrepresented codon pairs of the MDV host, the chicken. A fully codon pair-deoptimized UL30 mutant could not be recovered in cell culture. The sequence of UL30 was divided into three segments of equal length and we generated a series of mutants with different segments of the UL30 recoded. The codon pair-deoptimized genes, in which two segments of UL30 had been recoded, showed reduced rates of protein production. In cultured cells, the corresponding viruses formed smaller plaques and grew to lower titers compared with parental virus. In contrast, codon pair-optimized and -randomized viruses replicated in vitro with kinetics that were similar to those of the parental virus. Animals that were infected with the partially codon pair-deoptimized virus showed delayed progression of disease and lower mortality rates than codon pair-optimized and parental viruses. These results demonstrate that CPBD of a herpesvirus gene causes attenuation of the recoded virus and that CPBD may be an applicable strategy for attenuation of other large DNA viruses.

## Author summary

Codon pair bias deoptimization (CPBD) enables highly efficient attenuation of viruses. In contrast to other methods, live attenuated virus vaccine candidates can be rationally designed and produced within days. The technique involves recoding of viral genes, while preserving their codon bias and amino acid sequence. Recoding increases the number of codon pairs that are statistically underrepresented in protein coding sequences of the viral host, and involves swapping of available synonymous codons. While CPBD has been used to attenuate RNA viruses, it has never been applied on large double-stranded DNA viruses, such as poxviruses, asfarviruses, or herpesviruses. We used CPBD to attenuate an oncogenic Marek’s disease herpesvirus. The mutant viruses contained a recoded UL30 gene, which encodes DNA polymerase. The UL30 was either codon pair-optimized, -randomized, or -deoptimized. Corresponding to the level of codon pair deoptimization, the mutant viruses had either a lethal phenotype or were severely attenuated in vitro and in vivo. Nonetheless, viral oncogenicity was not completely eliminated. Virus with codon pair-optimized UL30 had characteristics of the parental virus in vitro and in vivo. The results of our study imply that CPBD might be an applicable strategy for attenuation of other herpesviruses and potentially other large double-stranded DNA viruses.

## Introduction

The attenuation by codon pair (bias) deoptimization (CPBD) has enabled rapid and highly efficient attenuation of a wide variety of RNA viruses, including *Enterovirus C* (poliovirus) [1], *Influenza A virus* (IAV) [2–4], *Human immunodeficiency virus type 1* (HIV-1) [5], *Human respiratory syncytial virus* [6], *Indiana vesiculovirus* [7], and *Dengue virus* [8]. Some of the recoded viruses have shown 100,000-fold attenuation in comparison to their virulent parental viruses, and have been successfully used as highly protective experimental vaccines in mice and ferrets in the case of IAV [3, 9]. In contrast to existing attenuation methods, CPBD-based vaccines can be designed within minutes and produced synthetically within days.

The attenuation by CPBD is based on the observation that some codon pairs are found in protein coding sequences significantly less or more frequently than expected. The attenuation by CPBD involves reshuffling of the available codons in viral genes with the goal to maximize the number of codon pairs that are underrepresented in the protein coding sequences in the respective host. The recoded gene encodes the same protein and has the same codon bias as the parental gene, but its codon pair bias is perturbed. As a result, the mutated virus is antigenically identical with the pathogenic parent, but its virulence is reduced or absent.

While the exact mechanism of attenuation by CPBD remains unknown, it is suggested that underrepresented codon pairs create unfavorable conditions for protein translation, modifications, or folding, which results in the decrease of protein production of recoded genes [2, 3]. Because codon pairs, which contain CpG and TpA dinucleotides at the codon pair boundary, are among the most underrepresented codon pairs in eukaryotes, CPBD also inadvertently and markedly increases the number of CpG and TpA dinucleotides in codon pair-deoptimized genes [10, 11]. An alternative theory proposes that the inadvertent change of dinucleotide frequencies is responsible for viral attenuation by making the recoded viral genes susceptible to recognition by the innate immune response [10, 11]. A recent study showed that the host zinc-finger antiviral protein (ZAP) could be the long-suspected and enigmatic antiviral factor [12], because it binds to CpG-rich RNA and targets them for degradation by the RNA exosome, thereby inhibiting viral replication [13]. Viruses have evolved effective countermeasures that block the antiviral responses mediated by ZAP [14, 15], and/or reduced the level of CpG dinucleotides in their genomes to become undetectable by ZAP.

The strategy of attenuation by codon pair deoptimization has been successfully employed in attenuation of a variety of RNA viruses [1–4, 6–9, 16, 17], but it has never been tested on large double-stranded (ds) DNA viruses, such as asfarviruses, poxviruses or herpesviruses. The goal of this study was to determine whether a large ds DNA virus, *Gallid alphaherpesvirus 2* (GaHV-2), also known as Marek’s disease virus (MDV), can also be efficiently attenuated by CPBD.

MDV is the causative agent of Marek’s disease (MD), a highly contagious lymphoproliferative and immunosuppressive disease of chickens. Under field conditions, most chickens are infected with MDV within first days of life and the virus causes up to 100% mortality in unvaccinated hosts. Live attenuated vaccines play a crucial role in controlling MD. All chickens that are commercially raised are vaccinated in-ovo or immediately after hatching to prevent MD. While vaccination prevents or reduces MD symptoms, it does not prevent super-infection with virulent MDV strains, and permissive vaccines in turn might lead to the selection of more virulent viruses. [18–20].

Before chickens became farmed in large complexes, MDV strains of mild virulence were causing only minor problems. Intensive farming practices provided MDV with the opportunity to infect large and naïve populations of chickens and evolve towards greater virulence [19, 21, 22]. During the last few decades, increasingly virulent MDV strains emerged and caused rampant disease and high mortality in flocks vaccinated with the best MD vaccines available [21, 23, 24]. Thus, despite 40 years of vaccine development, MD still jeopardizes poultry and egg production on a world scale. One of the most important goal of current MD research, therefore, is the development of a vaccine superior to the gold standard, CVI988-Rispens [22].

To determine whether CPBD is a suitable approach for MDV attenuation, we recoded UL30 that encodes the catalytic subunit of the viral DNA polymerase (Pol). The UL30 ORF was codon pair- optimized, -randomized, or -deoptimized. Corresponding to the level of codon pair deoptimization, the mutant viruses had either a lethal phenotype, or were severely attenuated in vitro and in vivo. Nevertheless, the oncogenic potential of the virus was not completely eliminated. In contrast, virus with codon pair-optimized UL30 formed bigger plaques than the parental virus in vitro, but was not more pathogenic than the parental virus in vivo. The results of this study imply that CPBD might be an applicable strategy for attenuation of other herpesviruses, and other large ds DNA viruses.

## Results

### Calculation of codon pair scores

Codon pair bias has been found in every species examined and was shown to differ between species [1, 11]. Because MDV infects domestic chickens, we hypothesized that MDV, in the process of co-evolution, might have adapted to codon pair bias of chicken to achieve optimal translation efficiency of its genes. To determine codon pair bias in chicken protein coding genes, we calculated a codon pair score (CPS) for each of the 3,721 codon pair combinations (61 × 61 codons, excluding the stop codons) using the method described by Coleman et al. [1]. A positive CPS value means that a given codon pair is found in chicken protein coding DNA sequences more often than it would be expected based on the individual frequencies of codons that form the given codon pair. Similarly, a negative CPS value means that a codon pair is underrepresented in the chicken ORFeome. When we compared the chicken CPS with the human CPS values [1, 11], we found out that the CPS of the two species are very similar (S1 Fig).

**Fig 1:**
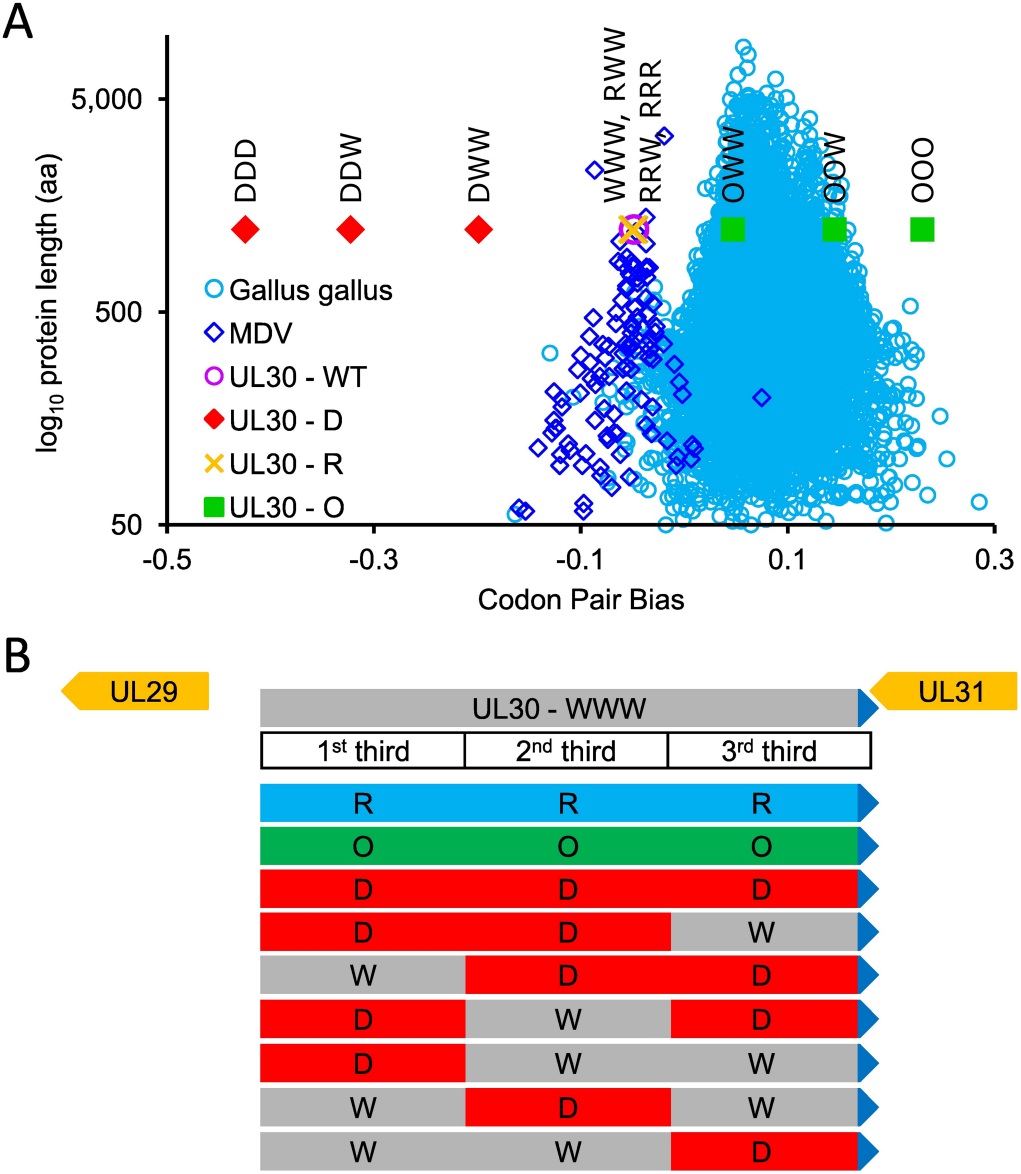
Distribution of codon pair bias (CPB) scores and recoding of the MDV gene UL30. **(A)** Distribution of calculated codon pair bias (CPB) scores of 15,762 predicted chicken, 112 MDV, and recoded MDV UL30 genes. Each light blue circle represents a calculated CPB score of a single chicken protein coding gene plotted against its protein length (amino acids). The arithmetic mean of all 15,762 CPB scores is 0.0755. The blue diamonds represent 112 predicted MDV protein coding genes. The pink circle represents original, wild type, labeled WWW, MDV UL30 gene. RWW, RRW and RRR (yellow crosses), which overlap each other and the parental UL30 represent calculated CPB scores of recoded MDV UL30 genes that have one, two or three segments of the ORF codon pairs-randomized. The red diamonds and green squares represent recoded UL30 genes that have either first, first two or all three segments of UL30 ORF codon pair-deoptimized (DWW, DDW, DDD) or optimized (OWW, OOW, OOO). **(B)** Structure of the MDV UL30 genomic region. UL30 encodes the catalytic subunit of the DNA polymerase, UL31 encodes a nuclear egress protein. UL30 and UL31 overlap at their 3’ ends by 98 nucleotides. UL30 is 3,663 nucleotide long and was divided into three equally long subsequences (1,221 nucleotides each), and each sequence was individually codon pair-optimized, -deoptimized, or -randomized. By recoding the individual MDV UL30 parts separately it is possible to generate different MDV UL30 mutants where only one (DWW, WDW, WWD), two (DDW, DWD, WDD), or all three UL30 segments (DDD) are recoded. The last 201 nucleotides of UL30 (blue triangles), which contain a polyadenylation signal and the overlapping coding sequences of UL31 were not altered by recoding.

Using the obtained chicken CPS, we calculated an average CPS, also labelled as codon pair bias (CPB) score, for each of the 15,762 predicted chicken protein coding sequences that we used in the analyses of CPS [11]. A negative CPB means that a coding sequence contains mostly under-represented codon pair combinations. We plotted each chicken gene’s calculated CPB score against its length in codon pairs to visualize the distribution of CPB scores of chicken genes (Fig 1A). The majority of chicken protein coding genes have positive CPB scores, and the mean of all 15,763 CPB scores is 0.076, which is almost identical with the mean CPB score of 0.075 of all human genes [1]. We used chicken CPS to calculate CPB of all predicted protein coding genes of MDV, and we observed that MDV genes had significantly lower CPB scores (median CPB -0.06) than the chicken genes, which suggests that encoding of MDV genes is not considerably influenced the codon pair bias of its host (Fig 1A).

### Algorithm for recoding genes to minimize, maximize or preserve the original CPB

We developed a program that uses the calculated chicken CPS and recodes a given protein coding sequence to a sequence with a desired CPB value. The recoding program reshuffles the synonymous codons of a given sequence to maximize (= optimization), minimize (= deoptimization) or preserve (= randomization) the CPB score of the sequence. Because only synonymous codons are swapped during recoding, the recoded sequence contains the same codon bias and encodes the same protein as the parental sequence. The algorithm also controls the free energy of folding RNA in a narrow range to prevent formation of large secondary structures, for example hairpins, as a consequence of reshuffling. It is important to keep the free energy of recoded genes in a narrow range to ensure that reduction of protein production is caused by codon pair deoptimization and not by extensive secondary RNA structures. We used the developed program to recode 112 predicted protein coding ORFs of MDV to minimize or maximize the CPB score (S2 Fig). The median CPB of parental, codon pair-deoptimized and codon pair-optimized genes was -0.06, -0.45 and 0.28, respectively.

### Recoding of the MDV UL30 gene

Our goal was to determine whether CPBD is an applicable strategy for attenuation of herpesviruses. We selected the very virulent RB-1B strain of oncogenic MDV as our model virus, and recoded gene UL30, which encodes the catalytic subunit of DNA polymerase (Pol), to study the effect of codon pair randomization, optimization and deoptimization on protein production, viral replication and attenuation. We recoded the UL30 gene, because DNA Pol is essential for virus replication in vitro and in vivo, and we expected that reduction or increase of DNA Pol protein production might change biological properties of the mutant viruses, which could be easily observable in cell culture and in vivo.

Because it was impossible to predict how strong an effect of codon pair deoptimization of UL30 on viral fitness would be, and because we conjectured that codon pair deoptimization of the entire UL30 gene might lead to a lethal phenotype, we recoded ORF UL30 such that we could produce, if needed, MDV mutants with different levels of codon pair deoptimization, optimization, or randomization. Therefore, we divided the UL30 coding sequence (3,663 nt) in silico into three equally long segments (1,221 nt). The segments were recoded separately–producing genes with three codon pair-optimized (OOO), -deoptimized (DDD) or -randomized (RRR) segments (Fig 1B). Because the coding sequences of the UL30 and the essential UL31 gene overlap at their 3’ ends, we recoded only the first 3,459 nucleotides (1,153 codons), and left the last 204 nucleotides of the UL30 ORF intact. As a result, only the first 1,017 nucleotides of the third UL30 segment were recoded (Fig 1B). The unaltered sequence contains the overlapping coding sequences (77 nucleotides) and the polyadenylation signal of the UL31 gene. Independent recoding of the UL30 segments enabled us to generate chimeric UL30 genes, in which any of the segments are recoded but the codon bias of the entire ORF is preserved. We constructed UL30 genes with one (DWW, WDW, and WWD), two (DDW, WDD, and DWD), or three (DDD) codon pair-deoptimized segments (Fig 1B).

The characteristics of the parental and recoded sequences are summarized in Table 1. As expected, reshuffling of synonymous codons introduced several hundred silent mutations in the recoded sequences. As observed previously [4, 10, 11], codon pair optimization reduced, and codon pair deoptimization markedly increased, the number of CpG dinucleotides in recoded sequences (Table 1). All sequences contain exactly the same codons, and therefore also same codon bias—which can be characterized by codon adaptation index (CAI) [25], but the order of codons in individual sequences is different. When two fully recoded sequences are compared to each other, on average 55% of all codons, which occupy the corresponding positions, are different. Because most of the synonymous codons share the first two and differ only in the third nucleotide, sequences are more similar to each other on the nucleotide than on the codon level. As expected, the parental, UL30-WWW and the UL30-OOO are the most alike on the nucleotide and codon level (Table 1). They contain the highest number of identical nucleotides (81.1%) and codons (51.5%) at the same positions. In contrast, UL30-OOO and UL30-DDD genes contain the least number of identical nucleotides (74.8%) and codons (34.6%) at the same positions (Table 1).

**Table 1:** Characteristics of parental and recoded UL30 genes.

Gene	CAI	CPB	CpG	Nucleotide identity (%)^a^	Codon identity (%)^b^	Silent mutations
UL30-WWW	0.78	-0.047	156			
UL30-OOO	0.78	0.228	94	81.1	51.5	693
UL30-RRR	0.78	-0.046	152	78.7	45.8	779
UL30-DWW	0.78	-0.196	213	92.6	80.6	270
UL30-WDW	0.78	-0.169	192	92.5	81.8	276
UL30-WWD	0.78	-0.148	182	93.9	84.3	224
UL30-DDW	0.78	-0.318	249	85.1	62.4	546
UL30-DWD	0.78	-0.297	239	86.5	64.9	494
UL30-WDD	0.78	-0.270	218	86.3	66.1	500
UL30-DDD	0.78	-0.419	275	79.0	46.7	770

All sequences contain exactly the same codons (same codon bias), but the order of codons in individual sequences is different (different codon pair bias). CAI–codon adaptation index; CPB–codon pair bias score; CpG–number of CpG dinucleotides; percentage of same nucleotides ^a^ or codons ^b^ that occupy the same position in the parental (UL30-WWW) and recoded genes.

### The effect of recoding on mRNA and protein levels

**Fig 2:**
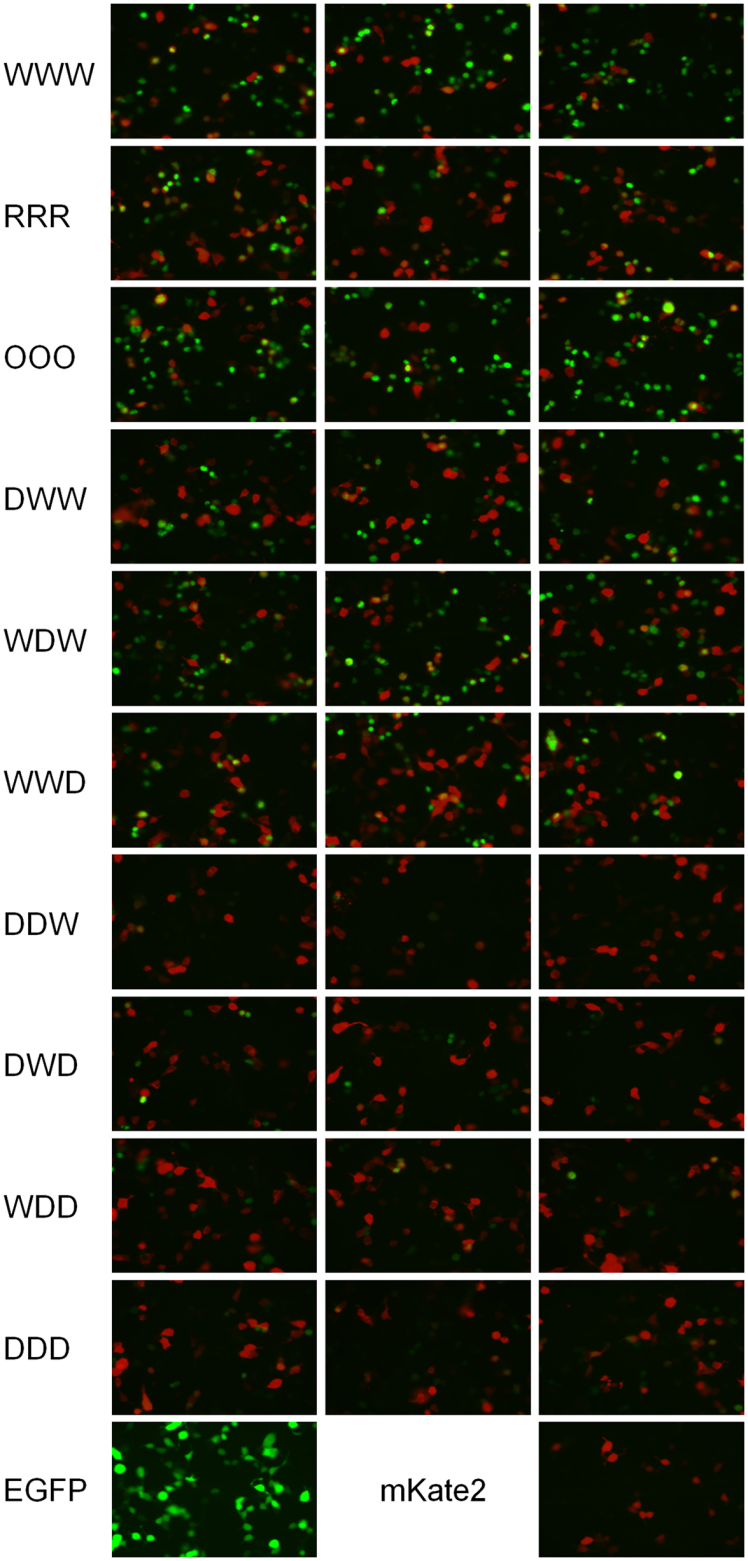
Protein production from differently recoded UL30 genes. Representative images of HEK 293T co-transfected with plasmid expressing mKate2 and recoded UL30-EGFP fusion genes. Note that EGFP in UL30-EGFP fusions can be found primarily in the nucleus because UL30 encoding DNA Pol contains a nuclear localization signal, but EGFP produced by the control plasmid (pEGFP-N1) is primarily present in the cytoplasm of transfected cells. Cells were imaged on a Zeiss Axiovert S100 microscope 24 h post transfection at 400-fold magnification.

To evaluate the effect of the recoding on UL30 protein production, we constructed expression plasmids pUL30-EGFP, in which UL30 expression was driven by the immediate-early promoter (IE) of *Human cytomegalovirus*, and UL30 genes were C-terminally tagged with EGFP. We transfected plasmids containing UL30-WWW, -RRR, -OOO, -DWW, -WDW, -WWD, -DDW, -DWD, -WDD and -DDD genes into DF-1, HEK 293T, HeLa and Vero cells. We obtained similar results in different cells lines (Fig 2 and S3–S6 Figs). While production of EGFP from the codon pair-randomized construct RRR was slightly reduced in comparison to the level of the parental WWW construct, the codon pair-optimized OOO construct produced more, and codon pair-deoptimized constructs produced less EGFP than the parental construct (Fig 2). In addition, the level of EGFP correlated with the level of codon pair-deoptimization of UL30: the genes with one codon pair-deoptimized segment produced only slightly less EGFP, but EGFP production was markedly reduced in genes that carried two or three codon pair-deoptimized segments.

**Fig 3:**
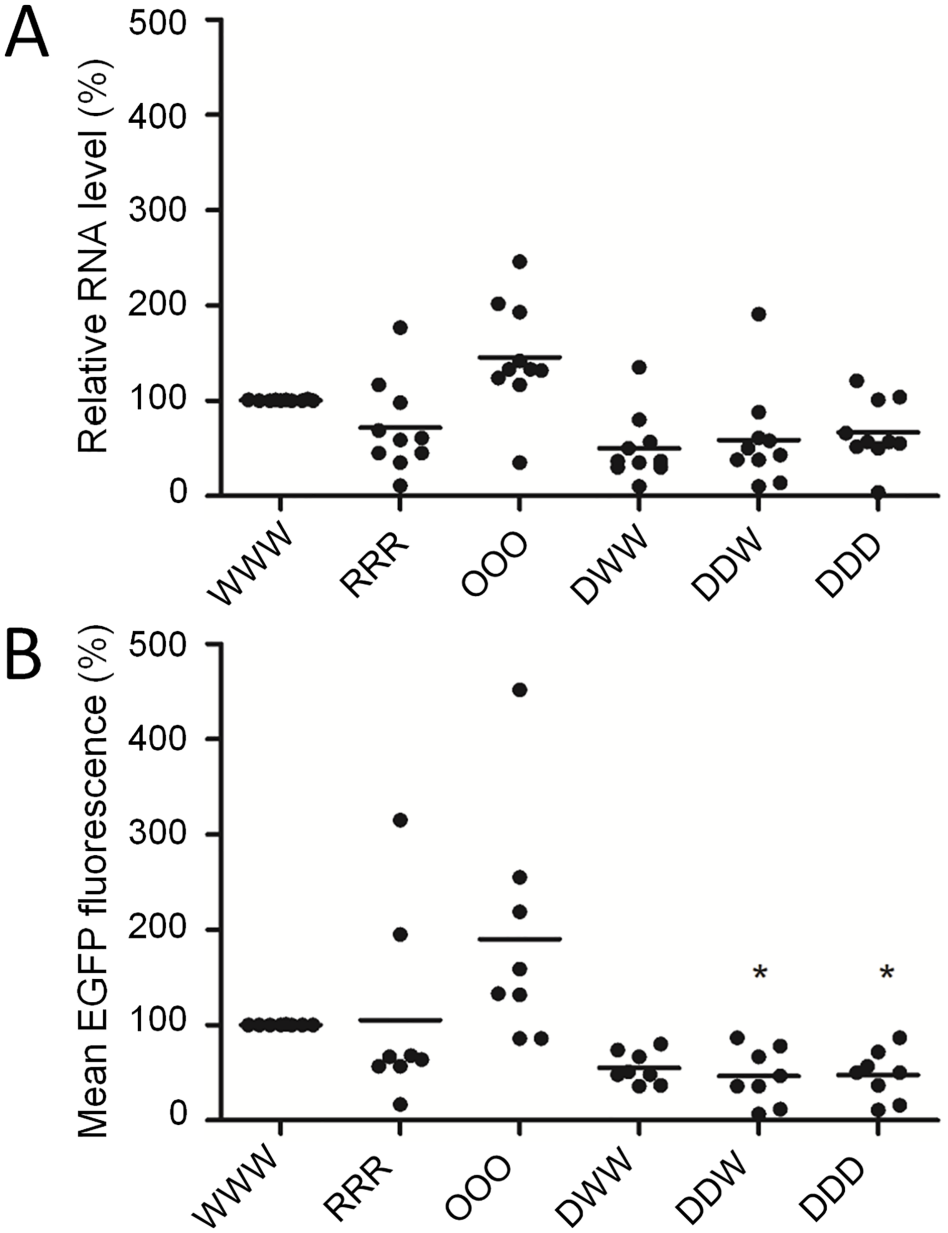
Quantification of RNA expression and protein production from the recoded UL30 genes. HEK 293T cells were transfected with dual expression plasmids pVITRO2-TagBFP-UL30-EGFP that carried differently recoded UL30 genes fused in frame with EGFP gene. 24 h post transfection RNA expression (A) from the recoded genes was quantified by qPCR, and protein production by flow cytometry (B). The UL30 RNA levels were normalized against the TagBFP levels. We used EGFP fluorescence as a reporter to quantify protein production of the fusion UL30-EGFP genes. The EGFP fluorescence was normalized against the TagBFP fluorescence. P-values were calculated using Kruskal-Wallis H test, * indicates P<0.05.

To quantify protein production of the parental and recoded genes, we constructed dual expression plasmids, in which expression of UL30-EGFP fusion gene and TagBFP was driven by two different promoters. The plasmids were transfected into HEK 293T cells and we analyzed mRNA and protein production from the parental and recoded genes 24 h after transfection. We used HEK 293T cells because they are highly transfectable, and because codon pair bias of human and chicken is highly similar (S1 Fig). qPCR analysis showed that codon pair-optimized gene produced slightly more, and codon pair-randomized and -deoptimized variants produced less mRNA than the parental UL30 gene, yet, the differences were not significant (Fig 3A).

We used the ratio of the EGFP to TagBFP fluorescence as a measure of protein production from different UL30 variants. Protein production from the UL30-OOO-EGFP was increased, and protein production from the codon pair-deoptimized genes was significantly reduced in comparison to the UL30-WWW-EGFP per amount of TagBFP (Fig 3B). These results confirmed that codon pair optimization had a positive effect, and codon pair deoptimization had negative effect on protein production of the recoded genes.

**Fig 4:**
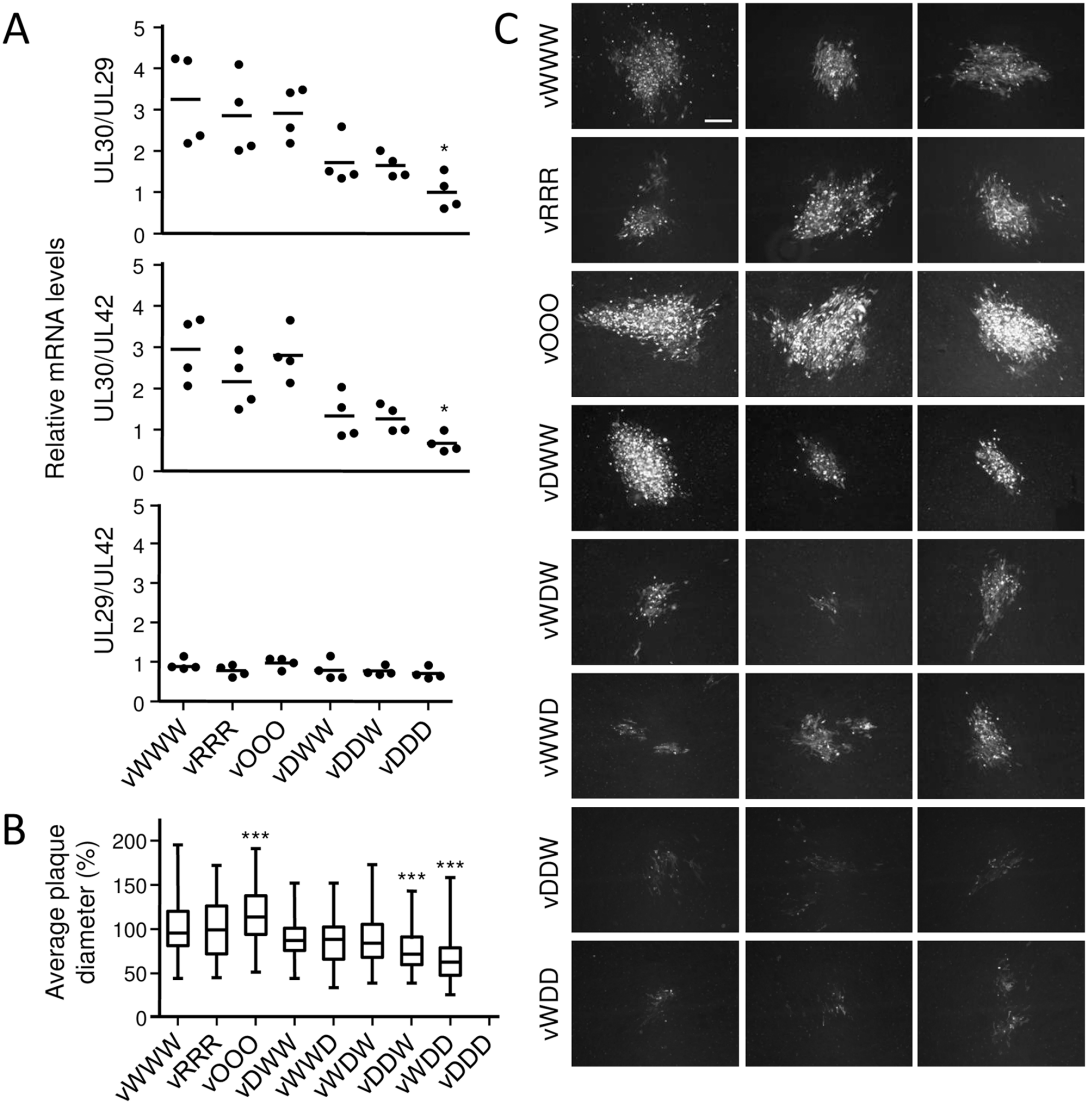
Characterization of recoded MDV UL30 mutants. (A) Effect of recoding on UL30 expression from the virus background. CEC were transfected with the parental or mutant BAC clones that carried differently recoded UL30 genes. 24 h post transfection RNA levels of UL29, UL30 and UL42 genes were quantified by qPCR. P-values were calculated using Kruskal-Wallis H test, * indicates P<0.05. (B) Plaque sizes of the parental and mutant viruses in CEC cells 6 days post infection. All images were taken at 100-fold magnification using an inverted fluorescence microscope. The box-plot displays the distribution of relative plaque diameter normalized against the average plaque diameter of the parental virus. P-values were calculated using one-way ANOVA Bonferroni’s multiple comparison test, *** indicates P<0.001. (C) Representative images of plaques formed by parental and mutant viruses. Plaque produced by the vOOO virus were larger, and plaques formed by the vDDW and vWDD viruses were markedly smaller than those formed by the vWWW virus. Scale bar, 200 μm.

To further investigate whether changes in UL30 RNA levels could be responsible for the observed differences in UL30 protein levels, we determined RNA expression from the parental and recoded UL30 genes during virus replication. Because MDV is strictly cell associated in vitro and in vivo, it is impossible to achieve synchronous infection of permissive cells by infection. To circumvent this inherent problem, we transfected viral BAC DNA into CEC and quantified the levels of viral UL29, UL30, and UL42 mRNA by qPCR 24 h post-transfection. We used the ratios of UL30 to UL29, and UL30 to UL42 to determine if recoding of UL30 affected RNA expression levels. We compared the expression levels of the UL30 to the levels of UL29 and UL42 because all three genes belong to the same kinetic gene expression class and represent early (β) genes. Furthermore, UL42 encodes the processivity factor of DNA polymerase and forms with UL30 the functional DNA polymerase. Quantification of RNA expression showed that codon pair deoptimization had a negative effect on RNA expression during virus replication, but only the UL30-DDD mutant produced significantly less UL30 RNA than the parental virus (Fig 4A).

### Construction and characterization of recoded viruses in tissue culture

We constructed MDV viruses in which the parental UL30 was replaced with the recoded genes. The mutants were made by 2-step Red *en passant* mutagenesis of pRB-1B, the infectious bacterial artificial chromosome (BAC) clone of MDV RB-1B [26]. In addition, from each mutant clone, we constructed a revertant by restoring the parental sequence of the UL30 region. The BAC mutants were examined by restriction fragment length polymorphism (RFLP) analysis, and sequencing of the mutated UL29-UL31 region.

Initially, we constructed BAC mutants with fully codon pair-randomized, -optimized and -deoptimized genes (pUL30-OOO, pUL30-RRR and pUL30-DDD). The BACs were transfected into CEC and we recovered parental vWWW, vRRR and vOOO viruses, but we failed to recover infectious progeny of the vDDD mutant despite five independent transfections and five successive blind passages of independent pUL30-DDD BAC clones. Because the vDDD revertant construct could be recovered, we concluded that the vDDD had a lethal phenotype because the level of codon pair deoptimization was too high.

We then generated additional mutants in which only one (pUL30-DWW, pUL30-WDW and pUL30-WWD) or two segments (pUL30-DDW and pUL30-WDD) of the UL30 were codon pair- deoptimized (Fig 1B). Mutant viruses were reconstituted by transfection of CEC, and we noted that the efficiency of reconstitution was variable among mutants. While 2 to 3 passages were necessary to observe a cytopathic effect (CPE) of the codon pair-deoptimized mutants, the CPE of the parental, vOOO, vRRR and revertant viruses could be readily observed immediately after transfection or in passage 1 after transfection.

Replication properties of mutant viruses in CEC were assessed by plaque size assays and multistep growth kinetics. As expected, viruses with two codon pair-deoptimized UL30 segments formed significantly smaller plaques than the parental vWWW virus. In contrast, mutants with only one segment deoptimized (vDWW, vWDW, vWWD), the randomized vRRR mutant and all revertant viruses formed plaques with sizes that were virtually identical to those of vWWW (Fig 4B). Unexpectedly, vOOO virus replicated more efficiently than the parental vWWW, as the plaques were bigger than those of the parental virus (Fig 4B).

The plaques formed by vDDW, vDWD and vWDD were not only smaller than those of the parental virus, but also differed in morphology (Fig 4C, S7 Fig). Plaques that were formed by the codon pair-deoptimized viruses had fewer infected cells and were interspersed by many uninfected cells. Infected cells in these plaques also stained less efficiently than infected cells of the parental virus.

**Fig 5:**
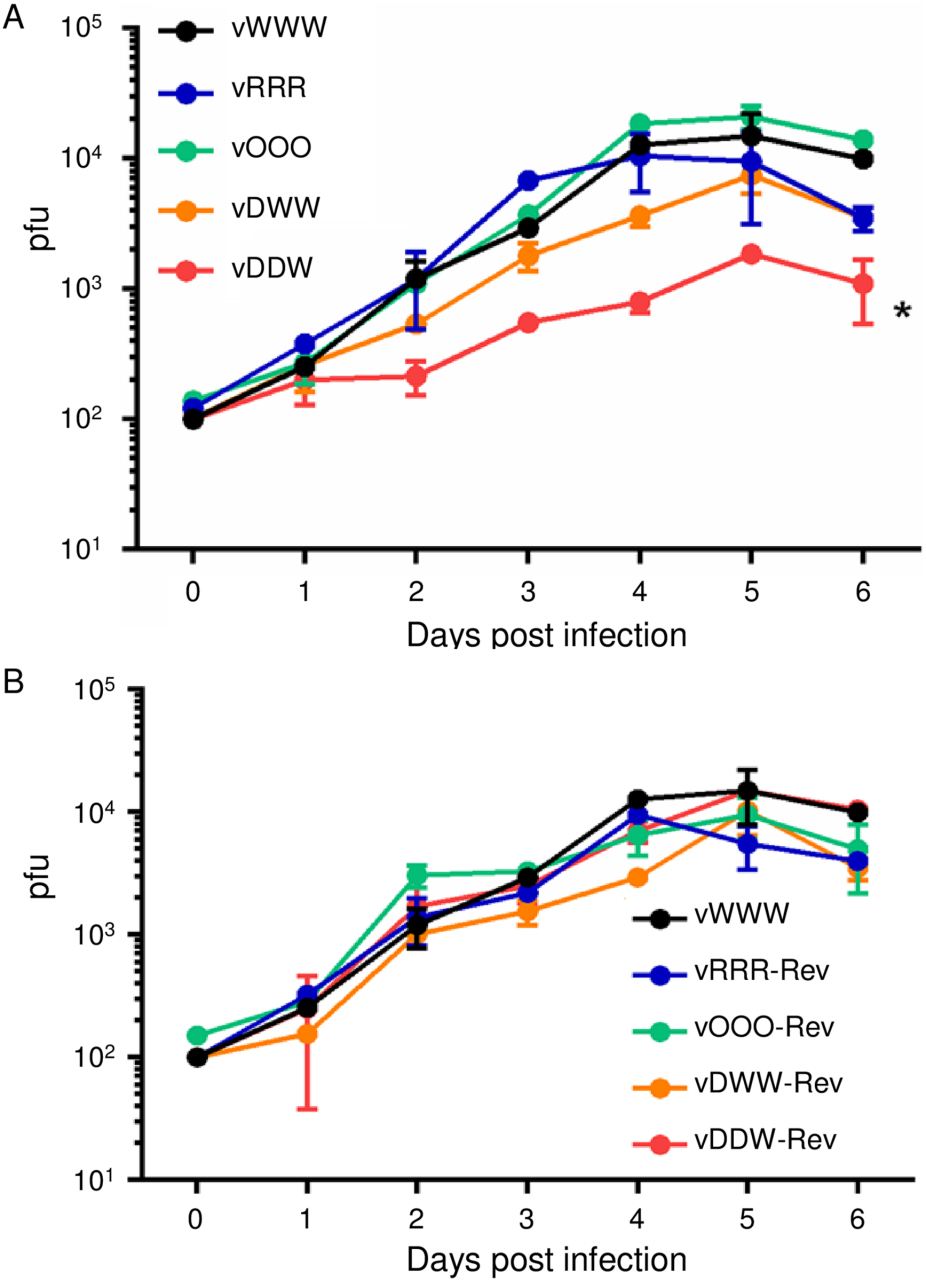
Multi-step growth kinetics of indicated viruses shown as mean and SEM. 1×10^6^ CECs were infected with 100 PFU and viral progeny was titered 1–6 days post infection. **(A)** Comparison of growth curves of the parental (vWWW) and mutant viruses (vRRR, vOOO, vDWW and vDDW); n = 6, Kruskal-Wallis H test, * indicates P<0.05. **(B)** Comparison of growth curves of the parental (vWWW) and revertant viruses (vRRR-Rev, vOOO-Rev, vDWW-Rev and vDDW-Rev); n = 6, Kruskal-Wallis H test, P<0.05.

Next, we determined the replication of vWWW, vRRR, vOOO, vDWW, and vDDW viruses in CEC by multi-step growth kinetics (Fig 5). All mutant viruses, with the exception of the vDDW (Fig 5A) and revertant viruses (Fig 5B), replicated with kinetics that were comparable to those of the parental virus. The vDDW grew to significantly lower titers than the parental virus (Fig 5A).

To test the genetic stability of recombinant viruses, we passaged the parental and mutant viruses sequentially in CEC at low multiplicity of infection. After 20 passages, we sequenced the UL30 region and determined plaque sizes of the passaged viruses. We did not detect any mutation in the recoded region, nor changes of the virus phenotype (S8A Fig) or virus replication properties (S8B Fig), confirming that recoded viruses were genetically stable.

### MDV with codon pair-deoptimized UL30 are attenuated in vivo

Because recoding influenced protein production of the UL30 genes, and because the vDDW replicated less efficiently in CEC than the parental virus, we hypothesized that recoded genes may also influence virus replication and tumorigenesis in vivo. To determine the pathogenic potential of the mutant viruses, we infected 1-day-old specific-pathogen-free chickens with parental vWWW, three mutants (vOOO, vDWW, vDDW), and the corresponding revertant (vOOO-Rev, vDWW-Rev or vDDW-Rev) viruses.

**Fig 6:**
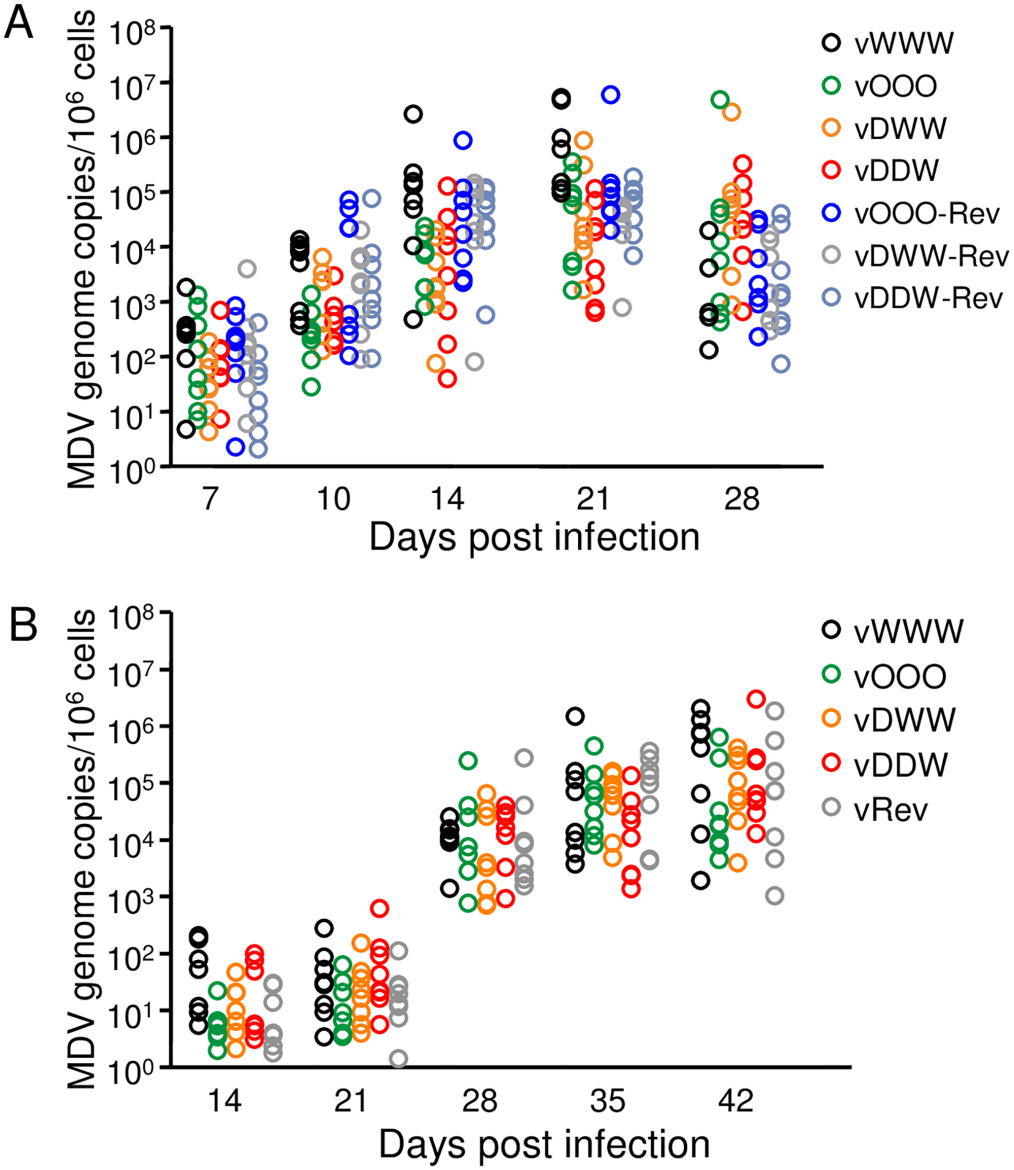
Replication of MDV in vivo. **(A)** Blood samples of chickens infected with the indicated viruses were taken at 4, 7, 10, 14, and 28 days post infection. **(B)** Contact chickens were sampled 14, 21, 28, 35 and 42 days p.i. Viral titers in the blood are shown as MDV genome copy numbers per 1×10^6^ cells of eight infected chickens per group. The detected viral loads are not statistically different among the groups, Kruskal-Wallis H test.

**Fig 7:**
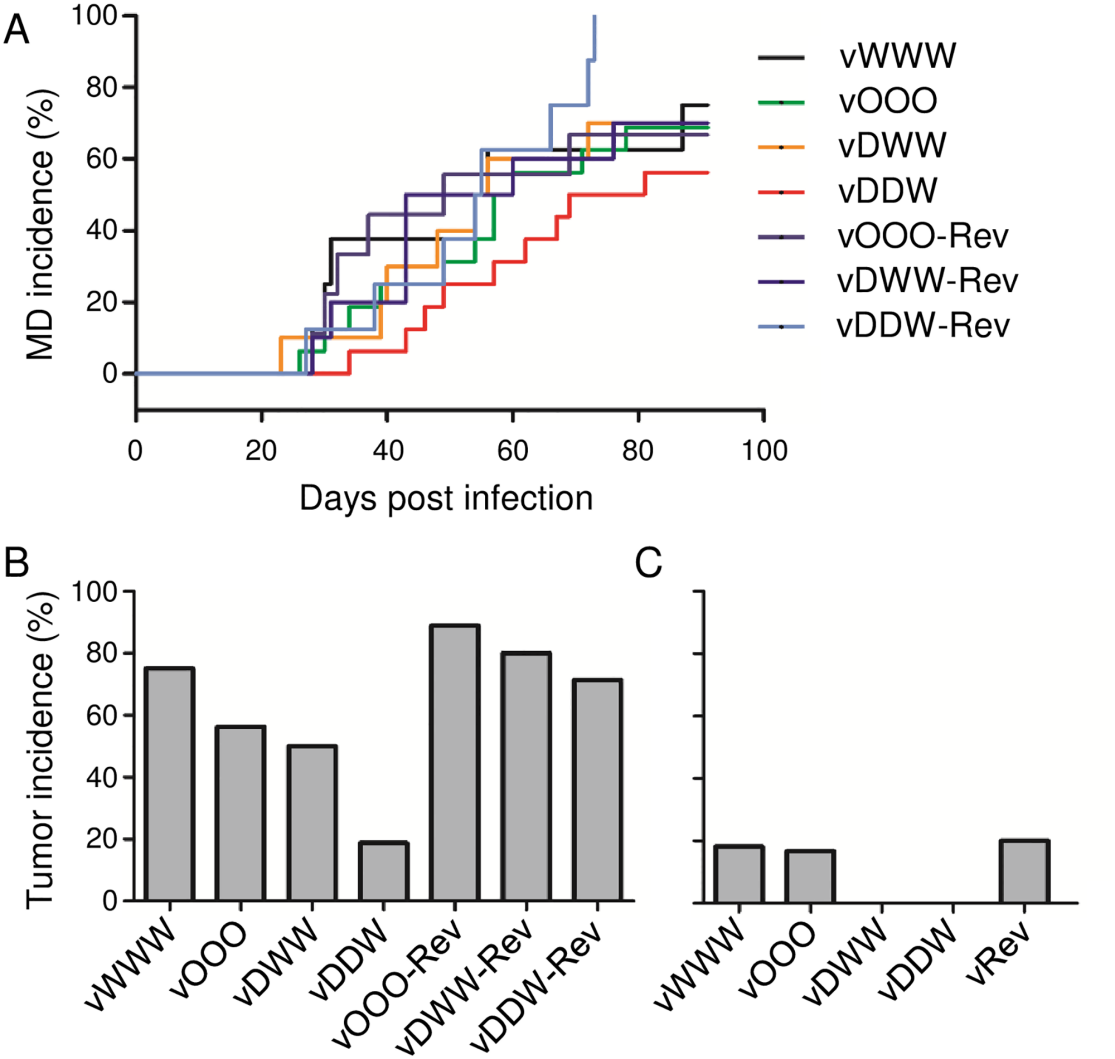
Codon pair deoptimization of UL30 impairs disease development and tumor formation in vivo. **(A)** MD incidence in chickens infected with the parental (vWWW), mutant (vOOO, vDWW, vDDW) and revertant (vOOO-Rev, vDWW-Rev, vDDW-Rev) viruses. Animals infected with the vDDW mutant showed lower MD incidence than those that were infected with the parental virus. Comparison of survival curves via Log-rank (Mantel-Cox) test did not identify statistical differences between the groups. Tumor incidence in infected chickens (**B**) and contact sentinel chickens that were housed together with the infected chickens (**C**). Chickens that were infected with three revertant viruses were housed together in one room and shared one group of sentinel chickens. Tumor incidence is shown as the percentage of animals per group. Differences in tumor incidence among the groups of infected chickens are statistically significant. Tumor formation was impaired in contact chickens that were housed together with vDWW or vDDW infected chickens. Statistical analysis was done by Chi-square test, P<0.0001.

To determine if recoding affected replication of the viruses in chickens we monitored levels of viral DNA in the peripheral blood by qPCR until 28 days post infection (p.i.). Analysis of the blood samples showed that birds infected with different viruses had similar viral loads at different times after infection, indicating that all viruses replicated within the host with similar kinetics (Fig 6A). However, animals that were infected with the vDDW viruses showed delayed progression of MD and lower mortality rates, albeit the differences were not statistically significant (Fig 7A).

At final necropsy, animals infected with vDWW and vDDW had fewer tumors than animals infected with parental virus vWWW (Fig 7B). In contrast to the parental and revertant viruses, contact birds that were housed together with chickens infected with vDWW and vDDW did not develop any tumors (Fig 7C).

We concluded from the results that MDV viruses with codon pair-deoptimized UL30, despite being severely attenuated in vitro, are still pathogenic for highly susceptible chickens, because codon pair deoptimization reduced the incidence of visceral tumors, but did not completely abrogate virus-induced tumorigenesis.

## Discussion

In the work presented here, we studied if codon pair deoptimization of an essential MDV gene can result in attenuation of the virus in its natural host. Herpesviruses, as many other large DNA viruses, are equipped with their own DNA replicase, which renders them independent from the host DNA replication machinery. The catalytic subunit of the herpesvirus DNA polymerase is a pivotal enzyme responsible for genome replication, and, thus, for successful transmission of genetic information from one generation to the next. As the DNA polymerase is essential for virus replication, we expected that alteration of DNA polymerase levels should result in phenotypic changes that would be easily observable in cell culture, for example in the form of reduced virus spread, and in vivo.

The exact molecular mechanisms that are responsible for attenuation by codon pair deoptimization remain unknown. However, it has been hypothesized that reduced translatability of codon pair-deoptimized genes might be the key factor responsible for attenuation of recoded viruses [1, 2]. To test this hypothesis, we examined several MDV mutants that carried codon pair-randomized, -optimized and -deoptimized UL30 variants. In line with our expectations, codon pair optimization or random reshuffling of codons in UL30 did not negatively affect the fitness of recoded viruses. In contrast, a virus that carried a fully codon pair-deoptimized UL30 displayed a lethal phenotype in cell culture. To identify why a fully codon pair-deoptimized virus was unable to replicate in cell culture, we constructed additional viruses with reduced levels of codon pair deoptimization. We constructed chimeric viruses in which approximately one-third (vDWW, vWDW, vWWD), or two-thirds (vDDW, vWDD) of UL30 were codon pair-deoptimized. Viruses of each of these two groups displayed similar phenotypes in vitro: while viruses with one codon pair-deoptimized UL30 segment replicated with parent virus-like kinetics, viruses in which two-thirds of the ORF was codon pair-deoptimized formed smaller plaques and replicated with significantly reduced kinetics when compared to the parent virus. These additional mutants also showed that none of the recoded segments itself, through potential negative effects on neighboring gene expression, was the cause of the observed lethal phenotype. The three types of codon pair-deoptimized mutants displayed a full spectrum of potential virus attenuation in vitro: viruses with one codon pair-deoptimized UL30 segment were very similar to the parental virus, the replication capacity of viruses with two codon pair-deoptimized segments was severely impaired, and a fully codon pair-deoptimized virus produced no viable progeny after transfection in susceptible cells. The results imply that the level of codon pair deoptimization correlated with the capacity of virus to replicate in vitro.

To better understand the effect of recoding on UL30 expression, we quantified RNA and protein production from the recoded UL30 genes in cells transiently transfected with expression plasmids but also during lytic infection of permissive cells in the virus background. The experiments showed that codon pair deoptimization had negative effect on RNA levels of UL30 after transient expression but also during virus replication (Figs 3A, 3B and 4A). In addition, we found a correlation between protein levels determined by flow cytometry and mRNA levels determined by qPCR. These results are in agreement with results from previous studies, which showed that codon pair deoptimization can lead to decreased mRNA levels [3, 4]. It is speculated that the reduction of mRNA levels could be caused by decreased expression of recoded genes, or increased mRNA degradation caused by the suboptimal, or stalled translation [4]. Still, because the differences in RNA levels measured in transient transfection experiments were not statistically significant (Fig 3A and 3B), and only the MDV mutant with the UL30-DDD gene produced significantly less UL30 RNA when compared to the parental gene (Fig 4A), we consider it unlikely that altered mRNA levels alone are responsible for the observed reduction in protein quantities and replication of viral mutants.

The recoded viruses were tested in vivo to determine if attenuation by codon pair deoptimization is a viable strategy for attenuation of MDV. In addition to the parental virus, we tested a codon pair- optimized, and two codon pair-deoptimized viruses (vOOO, vDWW and vDDW, respectively, Fig 7). From the tested viruses, only the virus with two codon pair-deoptimized segments, vDDW, exhibited significantly reduced replication kinetics in vitro when compared to the parental virus. Surprisingly, qPCR showed that all tested mutant viruses replicated in the natural host, and the level of replication was similar among different mutants (Fig 6A). As we predicted based on our in vitro data, animals that were infected with the recoded virus vDDW developed fewer tumors, and showed delayed progression of MD in comparison with the parental virus; yet, surprisingly, the overall mortality at 90 days p.i. was similar to chickens infected with the parental wild type virus. Despite the clear differences in MD progression and tumor formation, the codon pair deoptimization of RB-1B UL30 alone was not sufficient to render the virus fully attenuated in vivo.

Our study showed that codon pair deoptimization did not restrict bird-to-bird transmission of recoded MDV. The recoded viruses were shed from the feather follicles, as evidenced by the detection of replication of recoded viruses in contact birds by qPCR (Fig 6B). Sentinel birds that were housed with vDWW- and vDDW-infected chickens did not develop tumors. Yet, we suspect that tumor formation was only delayed in these animals, because vDWW and vDDW viruses replicated efficiently in contact chickens (Fig 6B).

Until now, the effect of codon pair deoptimization has been studied only in RNA viruses, which have relatively small genomes and a relatively small number of protein coding genes. As a result, codon pair deoptimization even of a single gene results in recoding of a relatively large proportion of total coding capacity of such viruses [1, 2, 4, 6, 8]. Previous studies with poliovirus [1], *Influenza A virus* [2] and *Dengue virus* [8] showed that recoding of multiple viral genes has a cumulative effect on virus attenuation. These studies also showed that the level of virus attenuation is not a function of the extent of viral genome deoptimization. Different genes contribute unequally to virus attenuation, and the contribution of each gene must be evaluated empirically. Consequently, it might be possible that codon pair deoptimization of a different essential MDV gene, or a combination of several genes, might result in satisfactory attenuation of MDV in vivo. An alternative speculation, namely that a codon pair deoptimization is an unsuitable method of attenuation for MDV, could be drawn based on the observation that a virus, which was severely impaired for replication in vitro (vDDW, Fig 5), still retained a high level of virulence in vivo (Fig 7). Because MDV establishes latency in T-cells as early as 7 days p.i. and that the latently infected T-cells become later neoplastically transformed, it allows us to speculate that no satisfactory level of virus weakening can be achieved by existing attenuation approaches–at least none that would abrogate the ability of MDV to transform infected cells and ultimately cause tumors. This would mean that a successful attenuation of MDV may be achieved, for example, by impairment of MDV replication by codon pair deoptimization and a deletion of the principal MDV oncogene meq.

However, because we managed to drastically reduce protein production of an essential gene without killing the virus, we expect that codon pair deoptimization is a suitable strategy for attenuation of large DNA viruses, preferably those that do not result in neoplastic transformation and for which pathogenesis relies mostly, if not exclusively, on lytic replication.

Only a limited number of studies explored the effect of codon pair optimization on viral properties [1]. Transient transfections showed that utilization of overrepresented codon pairs boosted protein production from the recoded, codon pair-optimized gene (Figs 2 and 3B). Interestingly, the virus carrying such a gene formed bigger plaques than the parental virus (Fig 4B and 4C), but replicated with parental-like kinetics in vitro (Fig 5), and was not more virulent than the parental virus in vivo (Fig 7). Thus, similar to codon pair-optimized poliovirus [1], recoding did not result in a virus that would replicate better than the parent in cell culture or in vivo. However, because recoding can lead to increased protein production, it remains to be determined if codon pair optimization, or other forms of protein recoding, could result in a virus that would be more virulent or pathogenic than the wild type.

## Materials and methods

### Ethics statement

All animal experimentation was done in full accordance with the EU legislation for the use of animals for scientific purposes (Directive 2010/63/EU) and German law (paragraph 8 Tierschutzgesetz). Animal housing, welfare and experimentation are under constant monitoring from an independent governmental institution. Animal experiments were approved by the Landesamt für Gesundheit und Soziales in Berlin, Germany (approval G0218-12). Fertile, specific-pathogen-free chicken eggs (Lohmann Tierzucht, Germany) were incubated in house, and 10-day-old embryos were used for production of primary chicken embryo cells (CEC).

### Recoding of viral genes

We used 15,762 predicted chicken protein coding genes (Gallus gallus, breed Red Jungle fowl, line UCD001, version 4.0) to quantify the level of underrepresentation/overrepresentation of each of the 3,721 possible codon pairs (61 × 61 sense codons) in the chicken ORFeome by calculating their codon pair scores (CPS) [1]. CPS is defined as the natural log of the ratio of the observed to the expected number of occurrences of a particular codon pair, and overrepresented codon pairs have positive CPS [1]. Using the calculated CPS we then calculated average CPS (CPB scores) for each of the 15,762 chicken and 112 MDV genes (S2 Fig).

We used the calculated chicken CPS to develop a computer program that can recode a given protein coding sequence to a new sequence with the desired CPB value. The program can reshuffle the available codons of a given sequence in order to preserve (codon pair randomization), minimize (codon pair deoptimization), or maximize (codon pair optimization) CPB of a given sequence. The recoding preserves codon bias, amino acid sequence, and the folding free energy of the recoded sequence.

Since there are many possibilities how a certain protein can be encoded, recoding of a gene to the maximal level of codon pair optimization/deoptimization is computationally difficult and time-consuming. An approximate, near-optimal solution of this problem, which is more than sufficient for our purpose, can be found quickly by heuristic or metaheuristic approaches. Our recoding program, similar to the algorithm of Coleman et al. [1], was designed to utilize simulated annealing, a fast metaheuristic algorithm, to locate a good approximation of the absolute CPB extreme [27].

Our algorithm also controls the free energy of folding RNA in a narrow range to prevent formation of extensive secondary structures (S9 Fig). It is important to keep the free energy of recoded genes in a narrow range in order to ensure that reduction of protein expression is caused by codon pair deoptimization and not by extensive secondary RNA structures. To ensure absence of extensive secondary structures in encoded RNA, we scanned the recoded sequences using the mFold program [28] exactly as described [1]. Briefly, from the coding sequences, we generated an array of overlapping fragments, which were 100 nucleotides long and had an 80 nucleotide overlap with each other. Then, we calculated the folding free energy for the produced fragments, and, when necessary, recoded all fragments that had free energy lower than -30 Kcal/mol by additional codon reshuffling to elevate the free energy of those particular regions. The final recoded sequences have a similar distribution of free folding energy over the length of the UL30 sequence and also a similar mean folding free energy (S9 Fig). The recoded sequences were synthesized (BioBasic, Canada) and cloned in the pUC57 vector (pUL30-RRR, pUL30-OOO or pUL30-DDD).

CAI of the parental and recoded genes was calculated based on the codon composition (codon bias) that is present in 15,762 predicted chicken protein coding genes [25].

### Cells and plasmids

Primary chicken embryo cells (CEC) were prepared from 10-day-old specific-pathogen-free embryos [29] and were cultured in minimal essential medium (MEM) with Earle’s salts, 100 U/ml penicillin, 100 μg/ml streptomycin and 1–10% fetal bovine serum (FBS). Human embryonic kidney 293T (ATCC CRL-3216), chicken embryo DF-1 (ATCC CRL-12203), human epithelial carcinoma HeLa (ATCC CCL-2) and African green monkey kidney Vero (ATCC CCL-81) cells were grown Dulbecco’s modified MEM (DMEM) with Earle's salts, 100 U/ml penicillin, 100 μg/ml streptomycin and 10% FBS.

To construct the pUL30-EGFP plasmids, the WT and recoded UL30 ORFs were cloned into pEGFP-N1 (Clonetech) between the NheI and AgeI restriction sites in frame with EGFP, so the DNA Pol is produced as fusion protein to the N-terminus of EGFP. To construct the pmKate2 plasmid the EGFP gene in pEGFP-N1 was replaced with mKate2 gene (Evrogen) encoding a far-red fluorescent protein.

To assess protein production from the parental WWW and recoded UL30 by flow cytometry we constructed pVITRO2-TagBFP-UL30-EGFP plasmids. The UL30 ORFs were fused C-terminally with EGFP and cloned into a dual expression plasmid pVITRO2-MCS (InvivoGen) under the control of human ferritin H/mouse elongation factor 1 promoter. The gene of blue fluorescent protein mTagBFP (Evrogen) was cloned into the second MCS, under the control of human ferritin L/chimpanzee elongation factor 1 promoter and was used for normalization of transfection efficiency.

### Generation of mutant viruses

The UL30 mutant viruses were generated based on the pRB-1B, an infectious BAC clone of the highly oncogenic MDV strain RB-1B [30], by two-step Red-mediated en passant mutagenesis in *Escherichia coli* as previously described [26]. Normally, during en passant mutagenesis, the target gene is replaced with the gene of interest in two steps. Here, the pRB-1B UL30 BAC mutants were generated in three steps. First the parental UL30 ORF was replaced with an ampicillin resistance marker, which was replaced in the second step with a recoded UL30 gene and a kanamycin selection marker. In the final step the kanamycin selection marker was removed by homologous recombination. The initial deletion of the parental UL30 was necessary to prevent undesired recombination between the recoded and parental variants, which are ~80% similar on nucleotide level. Primers used for the construction of the mutant and revertant BAC clones are shown in Supplementary data (S1 Table). The MDV BAC clones were analyzed by RFLP analysis, PCR and DNA sequencing of the target region.

To recover infectious viruses the purified BAC DNA and a Cre recombinase expression vector (pCAGGS-NLS/Cre) were co-transfected into CEC with polyethyleneimine [31]. Briefly, 1 μg of BAC DNA was diluted in 100 μl of serum-free MEM, mixed with 10 μl of 2 mg/ml linear polyethylenimine (MW = 25,000), incubated at room temperature for 30 min, and added to CEC grown to 80% confluency in a well of a 6-well plate. After 4 h of incubation at 37°C, the transfection mixture was removed and replaced with fresh medium. The expression of Cre recombinase ensured that BAC cassette flanked by loxP sites was efficiently removed from the infectious clones. The removal of the BAC vector from the MDV genome was confirmed by PCR as described previously [32]. Viruses were titrated on fresh CEC and viral stocks were stored in liquid nitrogen. Viral DNA was isolated from the infected cells with the method of Hirt [33].

### Serial passage of MDV

MDV was passaged serially in 100 mm dishes. In each new passage 5 × 10^6^ freshly seeded CEC were infected with 1,000 PFU of MDV, which corresponds to a multiplicity of infection of 0.0002.

### Multi-step growth kinetics and measurement of plaque areas

Multi-step growth kinetics were conducted as described previously [34]. Briefly, CEC (1×10^6^) seeded in a well of a 6-well plate were infected with 100 PFU of each virus. All infections were performed in duplicates. Cells were trypsinized 1, 2, 3, 4, 5 and 6 days post infection and 10-fold serial dilutions were inoculated onto fresh CEC. Plaques were stained (see below) and counted six days post infection.

To analyze cell-to-cell spread of viruses plaque areas were determined as described [35]. Briefly, CEC (1×10^6^) were mixed with infected CEC containing 50 PFU and seeded into a well of a 6-well plate. After 6 days, plaques were visualized by indirect immunofluorescence (see below). For each virus, images of 90 randomly selected plaques were taken at 100-fold magnification. Plaque areas were measured using ImageJ software (http://rsbweb.nih.gov/), from which, assuming that ideal plaques would have a circular shape, plaque diameters were calculated. Plaque diameters were plotted and analyzed using GraphPad Prism 7.02. Plaque sizes were determined in three independent biological experiments in double-blind fashion.

### Quantification of RNA expression

Expression plasmids pVITRO2-TagBFP-UL30-EGFP were transfected into subconfluent HEK 293T cells grown in 6-well plates with Lipofectamine 2000 (Thermo Fisher Scientific) in duplicates. Total RNA was isolated 24 h post transfection with RNAeasy kit (Qiagen). DNA was removed with DNaseI for 30 min at 37°C and 1 μg of RNA was reverse transcribed into cDNA with MMLV reverse transcriptase and random hexamers (Thermo Fisher Scientific). The cDNA was quantified by qPCR using the SYBR Green I system in an AB StepOnePlus Real-time PCR system (Thermo Fisher Scientific). The copy numbers were determined based on calibration curves generated for both genes based on known concentrations of pVITRO2-TagBFP-UL30-EGFP plasmid which was used as a standard.

To assess transcription of recoded UL30 genes in the viral background 1 μg of BAC DNA was transfected in duplicates into CEC grown in 6-well plates using polyethylenimine as described above. To synchronize infection of permissive cells the transfection mixture was incubated with cells only for 30 min. RNA was isolated 24 h post transfection as described above. The gene-specific primers and probes were used to quantify the viral transcripts of UL29, UL30 and UL42 genes (S1 Table) using the Luna Universal One-Step RT-qPCR Kit (NEB). The copy numbers were determined from the calibration curves generated for all three genes based on known concentrations of pRB-1B.

### Indirect immunofluorescence

Infected cells were fixed with 2% paraformaldehyde, permeabilized with 0.1% Triton-X 100, and blocked with 3% bovine serum albumen (BSA) in PBS. The cells were then incubated with anti-MDV chicken serum (dilution 1:2000) for 1 h, and then with goat anti-chicken IgG-Alexa Fluor 488 secondary antibody (dilution 1:2000, Invitrogen) for 45 min to visualize plaques. Images were taken at 100- and 200-fold magnification using an inverted fluorescence microscope (Axiovert S100, Zeiss).

### Protein quantification

To analyze protein production from recoded UL30 variants (WWW, RRR, OOO, DWW, WDW, WWD, DDW, DWD, WDD and DDD) DF-1, HEK 293T, HeLa or Vero cells were contransfected with 500 ng of pUL30-EGFP and 500 ng of pmKate2 plasmid. As a control pmKate2 and pEGFP-N1 were transfected alone. Images were taken 24 h post transfection at 200- and 400-fold magnification using an inverted fluorescence microscope (Axiovert S100, Zeiss).

Protein production was quantified by using a dual expression pVITRO2-TagBFP-UL30-EGFP plasmids. Plasmids were transfected into HEK 293T cells. 24 h post transfection cells were washed twice with PBS and resuspended in FACS buffer. Fluorescence of TagBFP and EGFP were measured in a CytoFlex flow cytometer (Beckman Coulter). Color compensation was done using a TagBFP and EGFP positive control to eliminate artifact due to the overlap of TagBFP and EGFP emission. Cell debris and duplets were excluded from further analyses.

### Animal experiments

One-day-old specific-pathogen-free Valo chickens (Lohmann Tierzucht, Germany) were infected intraperitoneally with 2,000 PFU of vWWW, vOOO, vDWW, vDDW, vOOO-Rev, vDWW-Rev or vDDW-Rev. Each experimental group was composed of sixteen infected chickens and eleven naïve chickens. The uninfected chickens were housed with the infected chickens to determine if mutant viruses were able to transmit via natural route by shedding. Chickens that were infected with three revertant viruses were housed together in one room, and therefore shared only one group of contact birds. Water and food were provided ad libitum. Animals were monitored for MD symptoms on a daily basis. After manifestation of clinical symptoms or termination of the experiment at 90 days after infection, animals were examined for tumors by necropsy.

### Quantification of MDV genome copies in chicken blood

Whole blood was taken by wing vein puncture from 8 randomly selected chickens of each group. Infected and contact chickens were sampled 4, 7, 10, 14, 21 and 28 days p.i. and 21, 28, 35 and 42 days p.i. respectively. Blood was taken from the same animals, and dead animals were not replaced. DNA was isolated out of the chicken blood using an E-Z96 96-well blood DNA isolation kit (Omega Biotek). The MDV genome copies in the chicken blood were quantified by qPCR using primers and probe for MDV gene ICP4 [35]. Number of cellular genome copies of the inducible nitric oxide synthase (iNOS) was used for normalization [35].

### Accession numbers

The recoded UL30 sequences are available under GenBank accession numbers MF671698 (UL30-RRR), MF671699 (UL30-OOO) and MF671700 (UL30-DDD), and also in the Supplementary data (S1 Appendix).

## Supporting information

S1 Fig Human and chicken protein coding sequences have similar codon pair bias. Each dot represents one of the 3,721 possible codon pairs (61 × 61 sense codons) and shows codon pair’s CPS in the human and the chicken. CPS were calculated using the available protein coding genes of the chicken (15,762) and the human (18,261).
(TIF)

S2 Fig Distribution of CPB scores of the original, codon pair-optimized and codon pair-deoptimized MDV RB-1B protein coding genes. The chicken CPB scores are shown as light blue circles, the WT, codon pair-optimized and -deoptimized MDV genes as shown as blue, green and red diamonds, respectively. The CPB scores are plotted against gene length in amino acids.
(TIF)

S3 Fig Protein production from differently recoded UL30 genes. Representative images of HEK 293T co-transfected with plasmid expressing mKate2 and recoded UL30-EGFP fusion genes. Note that EGFP in UL30-EGFP fusions can be found primarily in the nucleus because UL30 encoding DNA Pol contains nuclear localization signal, but EGFP produced by the control plasmid (pEGFP-N1) is primarily present in the cytoplasm of transfected cells. Cells were imaged 24 h post transfection at 400-fold magnification.
(TIF)

S4 Fig Protein production from differently recoded UL30 genes. Representative images of HeLa co-transfected with plasmid expressing mKate2 and recoded UL30-EGFP fusion genes. Cells were imaged 24 h post transfection at 400-fold magnification.
(TIF)

S5 Fig Protein production from differently recoded UL30 genes. Representative images of DF-1 co-transfected with plasmid expressing mKate2 and recoded UL30-EGFP fusion genes. Cells were imaged 24 h post transfection at 400-fold magnification.
(TIF)

S6 Fig Protein production from differently recoded UL30 genes. Representative images of Vero co-transfected with plasmid expressing mKate2 and recoded UL30-EGFP fusion genes. Cells were imaged 24 h post transfection at 200-fold magnification.
(TIF)

S7 Fig Representative images of plaques formed by parental and mutant viruses. Plaques of the parental and mutant viruses in CEC 6 days post infection. Images were taken at 200-fold magnification. Scale bar, 100 μm.
(TIF)

S8 Fig Plaques formed by the viruses that were passaged 20 times in cell culture. **(A)** Representative images of plaques formed by the parental and mutant viruses that were passaged 20 times serially at low multiplicity of infection in cell culture. Scale bar, 200 μm. **(B)** Serial passaging of viruses has not altered phenotype of mutant viruses. Similar to the viruses from early passages, plaque produced by the vOOO virus were larger, and plaques formed by the vDDW virus were smaller than those formed by the vWWW virus. The box-plot displays the distribution of relative plaque diameter normalized against the average plaque diameter of the parental virus. P-values were calculated using one-way ANOVA Bonferroni’s multiple comparison test, * indicates P<0.05.
(TIF)

S9 Fig Folding free energy (ΔG) of the parental and the recoded UL30 genes. ΔG of the RNA encoded by the codon pair-optimized (OOO), -deoptimized (DDD) and -randomized (RRR) UL30 is similar to parental (WWW) UL30 gene. ΔG was calculated for 100 bp sequences that had a 80 bp overlap with each other. ΔG of any 100 bp fragment derived from the three recoded genes is not lower than -30 Kcal/mol. The parental and recoded sequences have similar mean ΔG: UL30-WWW = -18.7, UL30-OOO = -18.7, UL30-DDD = -18.4 and UL30-RRR = -18.6.
(TIF)

S1 Table List of primers used for the construction of pRB-1B BAC clones and qPCR analysis. (DOCX)

S1 Appendix Sequences of the recoded UL30 genes. The coding sequence of the parental UL30 gene was divided into three equally long segments (1,221 bp), and each segment was recoded independently. The second 1,221 bp UL30 segment is in lowercase. The last 201 bp of the coding sequence that were not subjected to recoding are underlined.
(DOCX)
