# Supplementary figures and images for: Attenuation of a very virulent Marek's disease herpesvirus (MDV) by codon pair bias deoptimization
Source: PLoS Pathog. 2018 Jan 29;14(1):e1006857. doi: 10.1371/journal.ppat.1006857 (PMC5805365; doi:10.1371/journal.ppat.1006857)

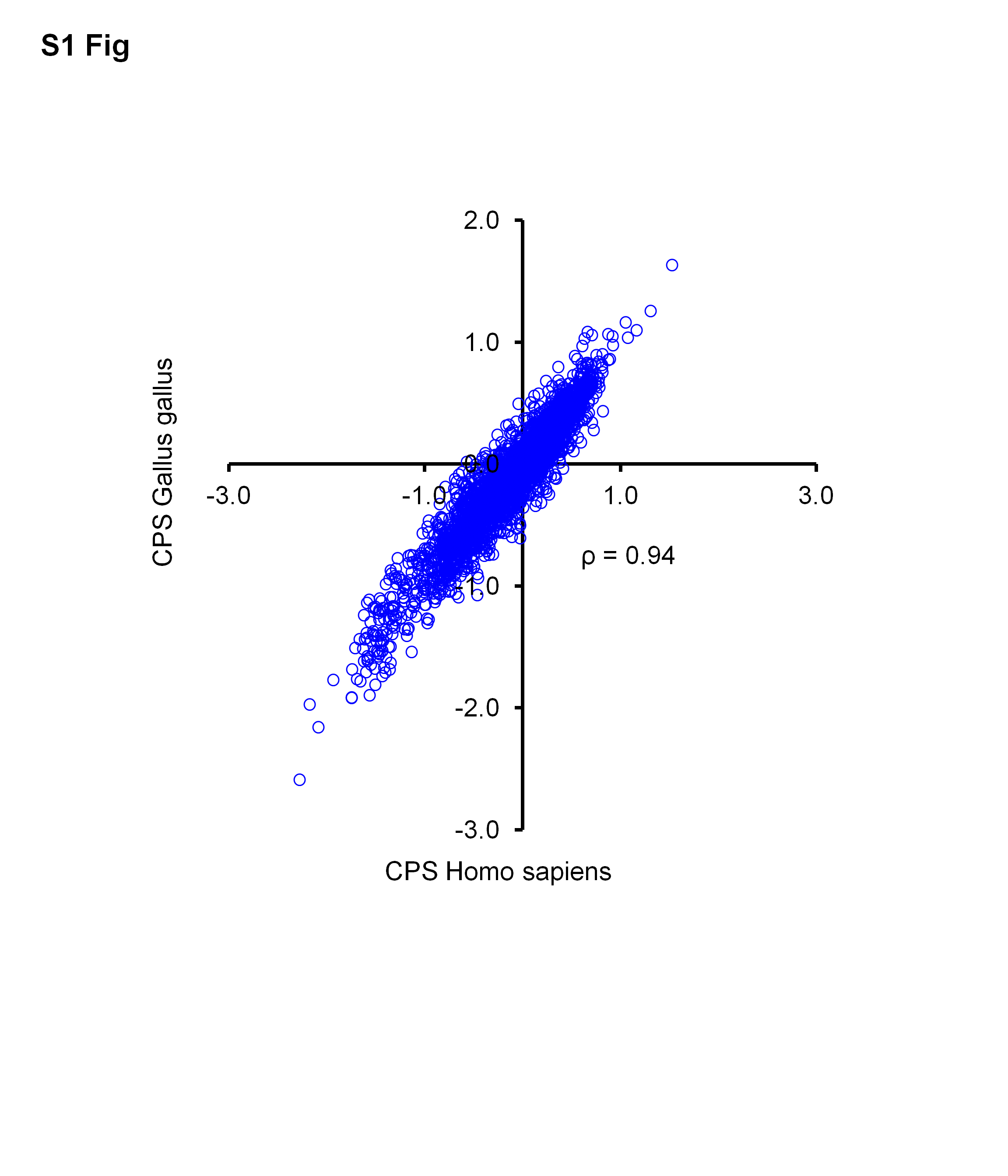

Supplement: S1 Fig — Each dot represents one of the 3,721 possible codon pairs (61 × 61 sense codons) and shows codon pair’s CPS in the human and the chicken. CPS were calculated using the available protein coding genes of the chicken (15,762) and the human (18,261). (TIF) [file ppat.1006857.s001.TIF]

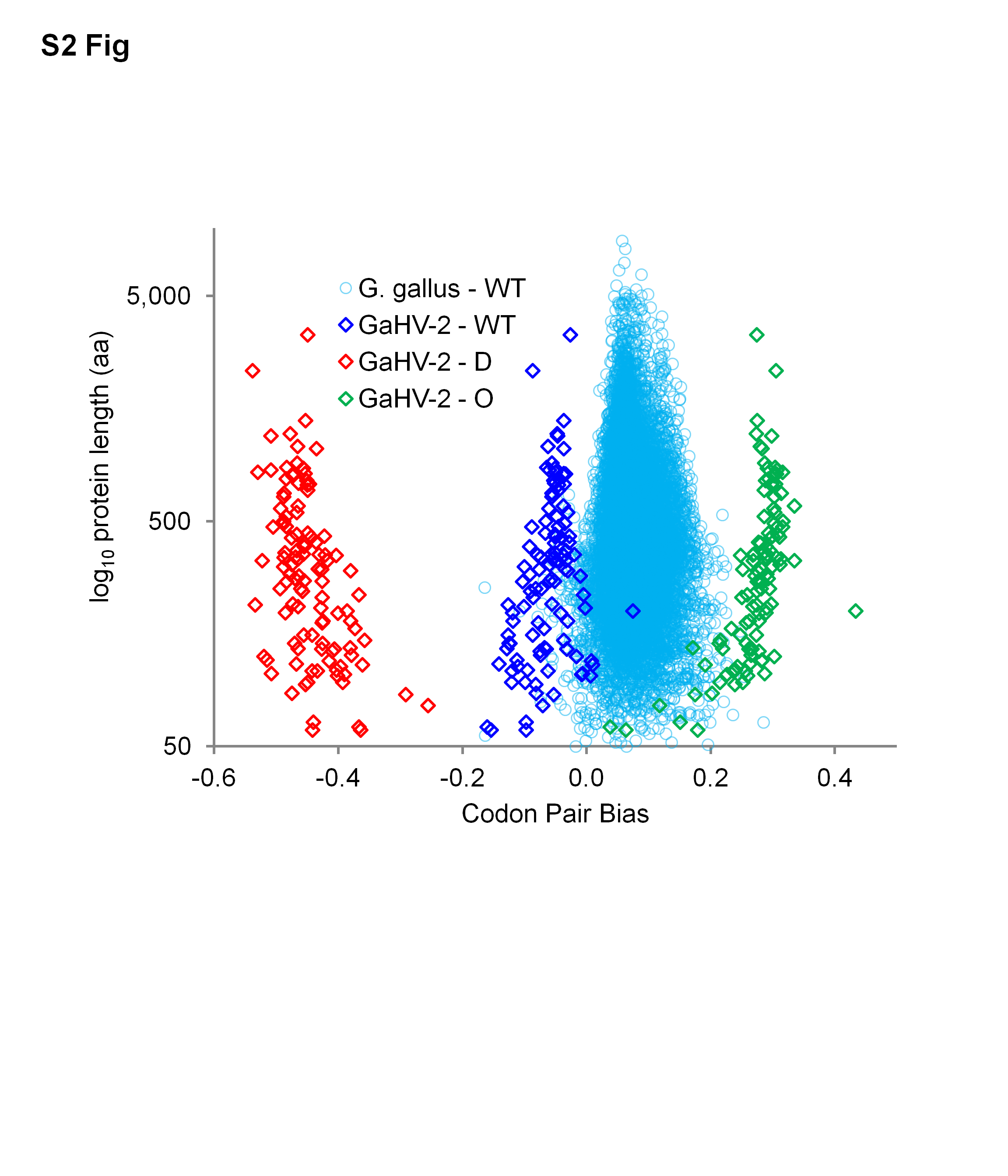

Supplement: S2 Fig — The chicken CPB scores are shown as light blue circles, the WT, codon pair-optimized and -deoptimized MDV genes as shown as blue, green and red diamonds, respectively. The CPB scores are plotted against gene length in amino acids. (TIF) [file ppat.1006857.s002.TIF]

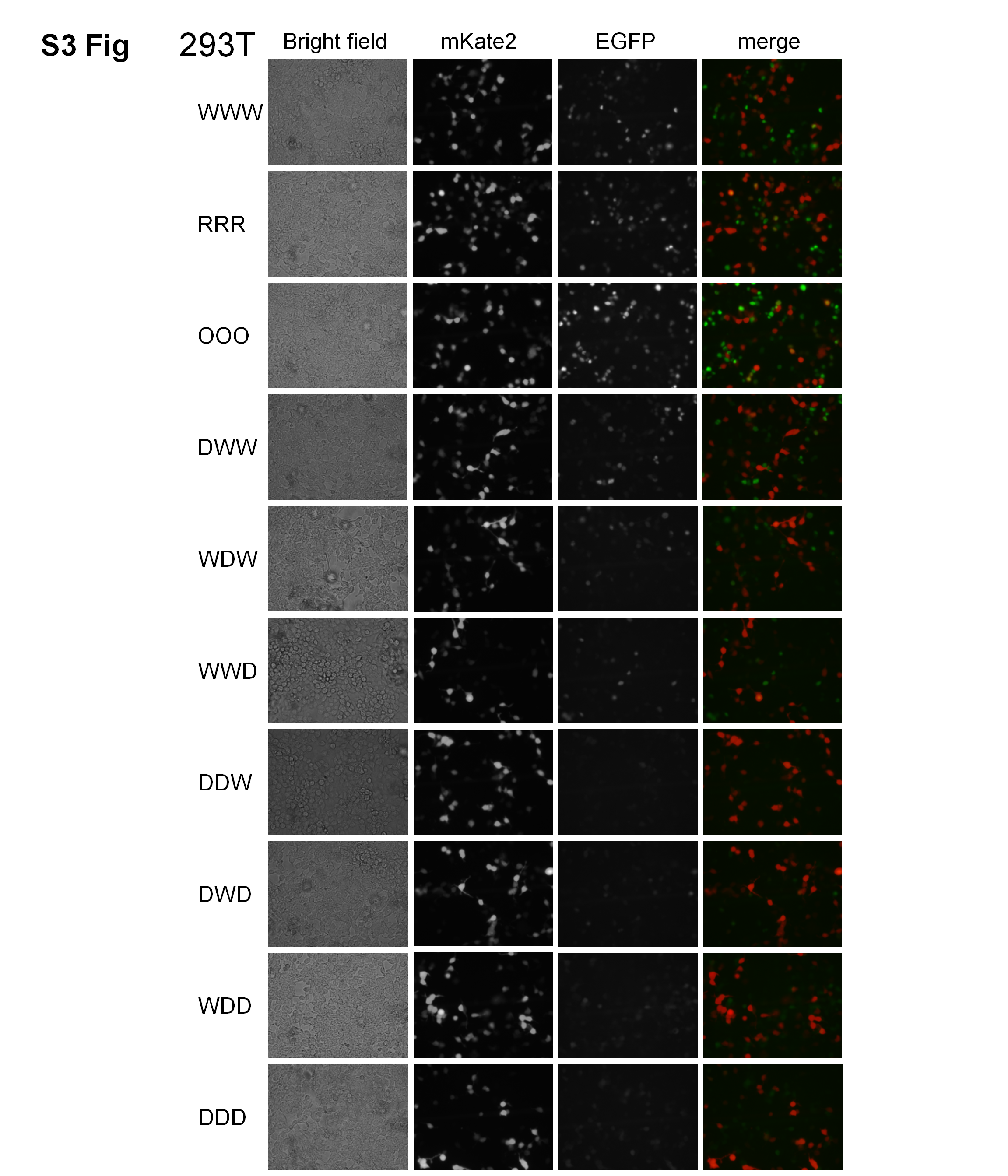

Supplement: S3 Fig — Representative images of HEK 293T co-transfected with plasmid expressing mKate2 and recoded UL30-EGFP fusion genes. Note that EGFP in UL30-EGFP fusions can be found primarily in the nucleus because UL30 encoding DNA Pol contains nuclear localization signal, but EGFP produced by the control plasmid (pEGFP-N1) is primarily present in the cytoplasm of transfected cells. Cells were imaged 24 h post transfection at 400-fold magnification. (TIF) [file ppat.1006857.s003.TIF]

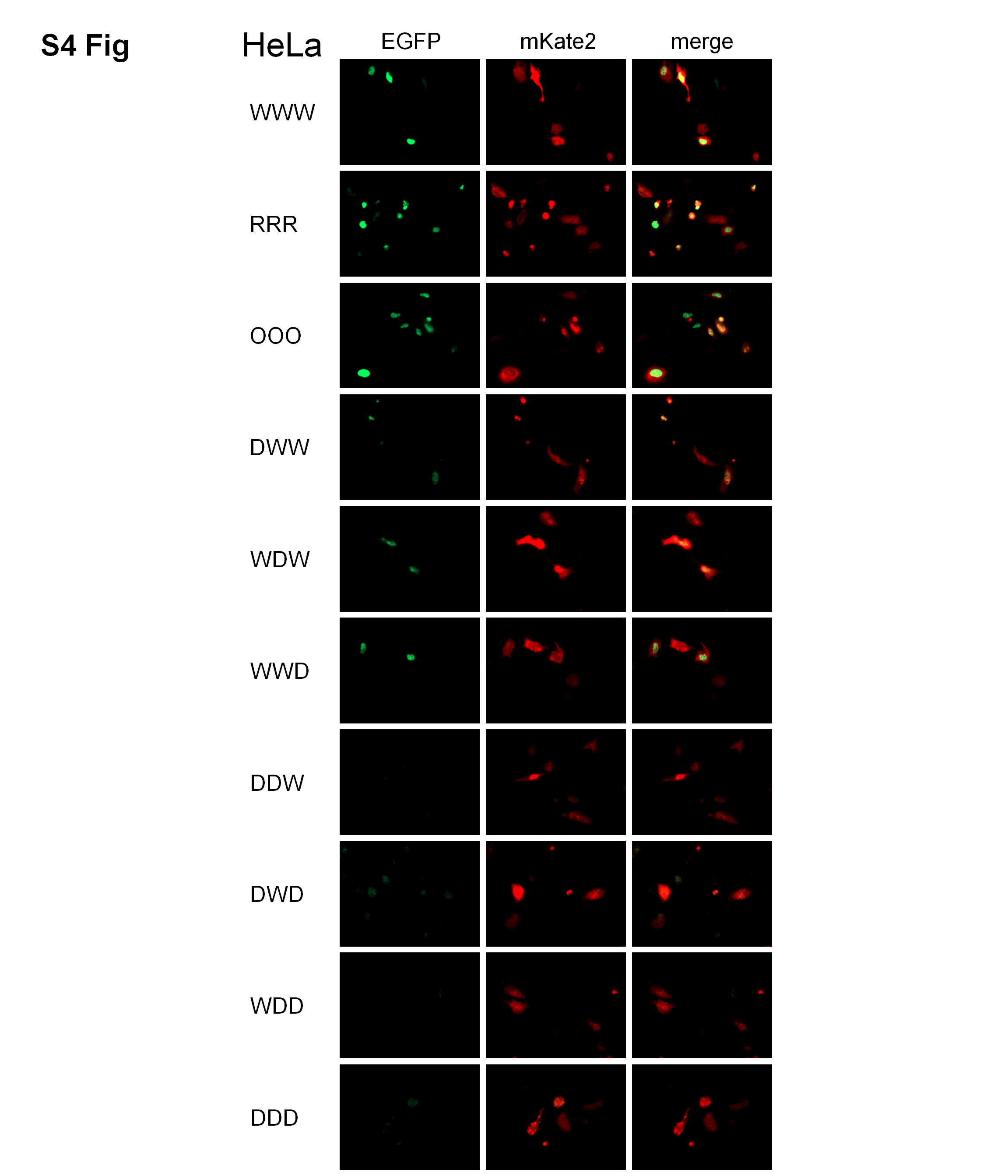

Supplement: S4 Fig — Representative images of HeLa co-transfected with plasmid expressing mKate2 and recoded UL30-EGFP fusion genes. Cells were imaged 24 h post transfection at 400-fold magnification. (TIF) [file ppat.1006857.s004.TIF]

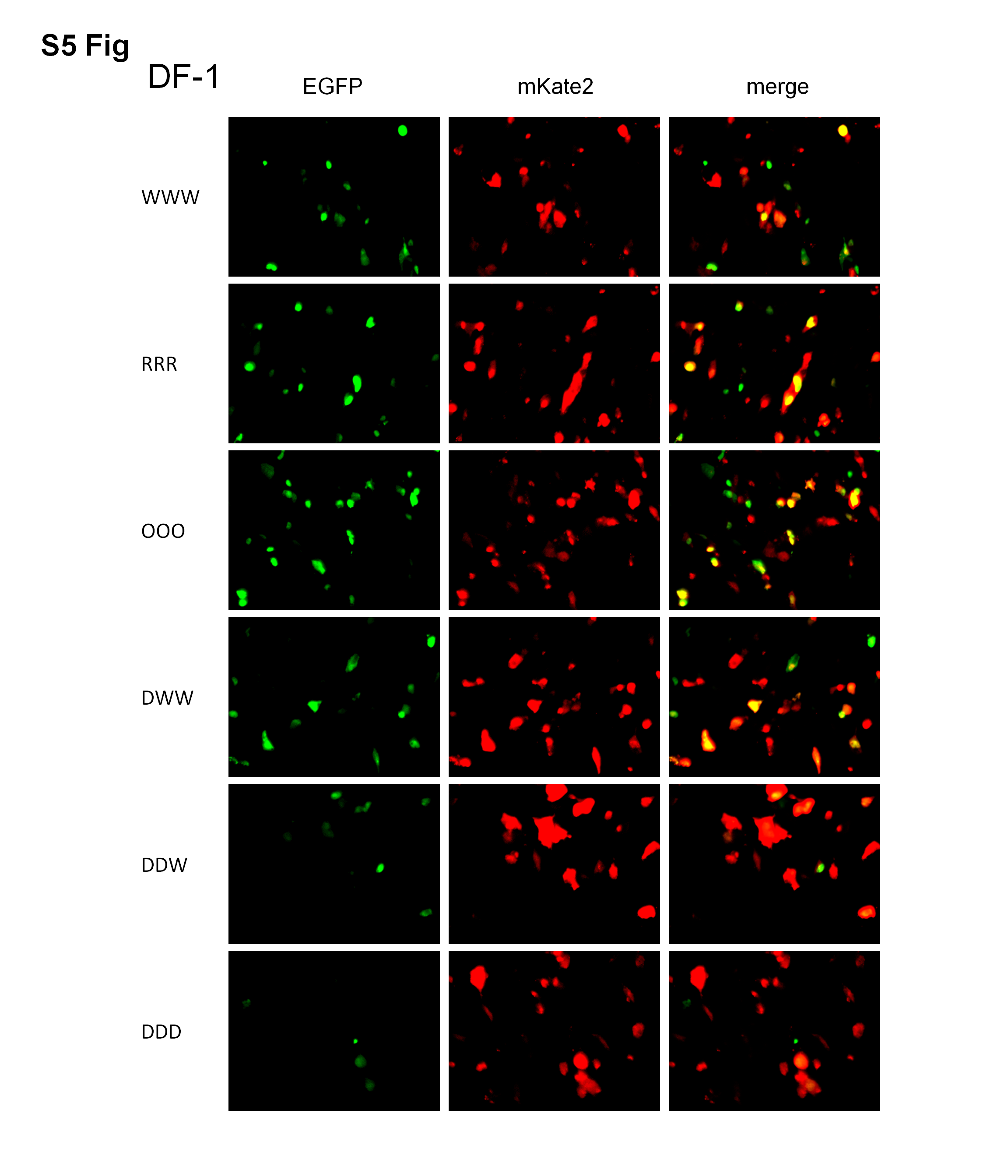

Supplement: S5 Fig — Representative images of DF-1 co-transfected with plasmid expressing mKate2 and recoded UL30-EGFP fusion genes. Cells were imaged 24 h post transfection at 400-fold magnification. (TIF) [file ppat.1006857.s005.TIF]

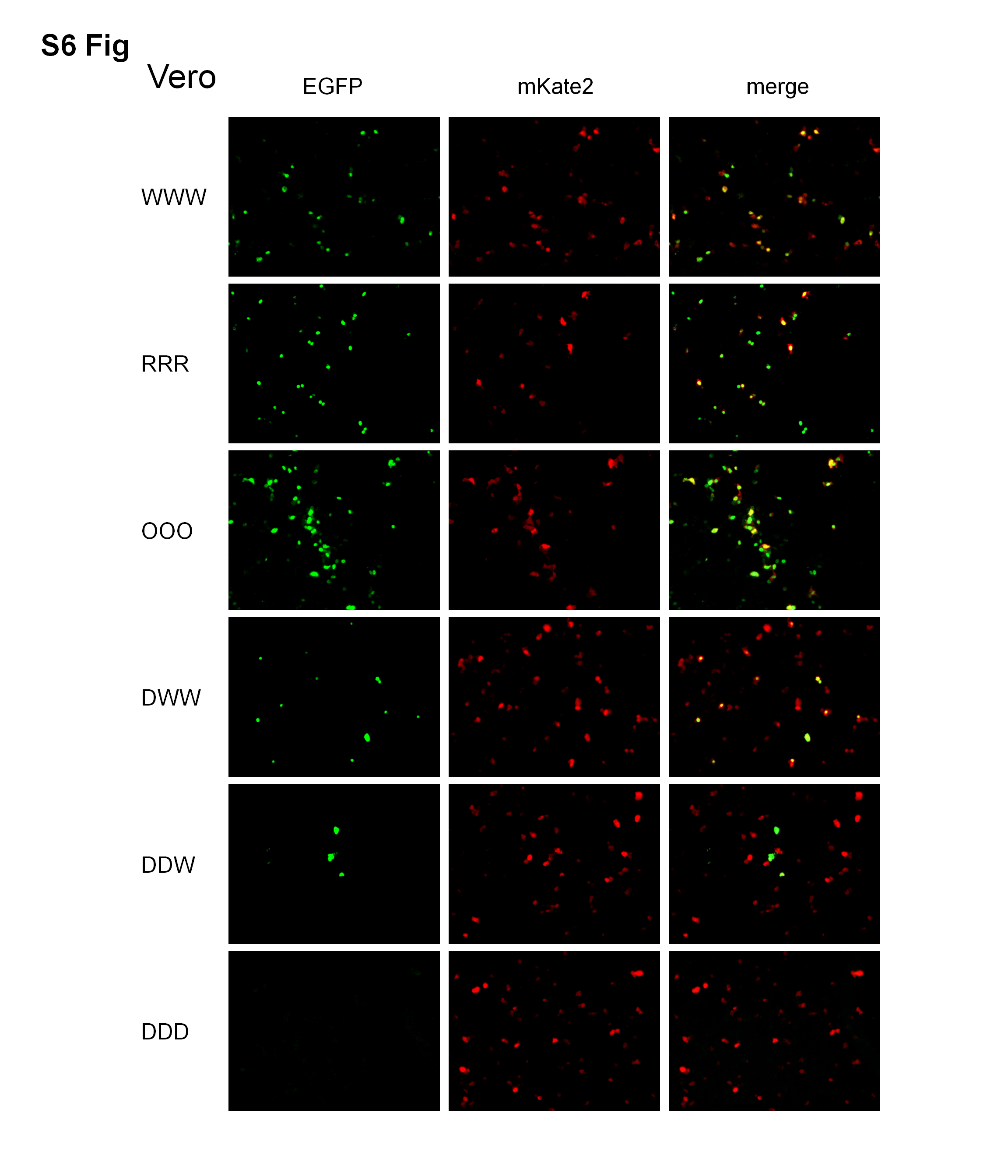

Supplement: S6 Fig — Representative images of Vero co-transfected with plasmid expressing mKate2 and recoded UL30-EGFP fusion genes. Cells were imaged 24 h post transfection at 200-fold magnification. (TIF) [file ppat.1006857.s006.TIF]

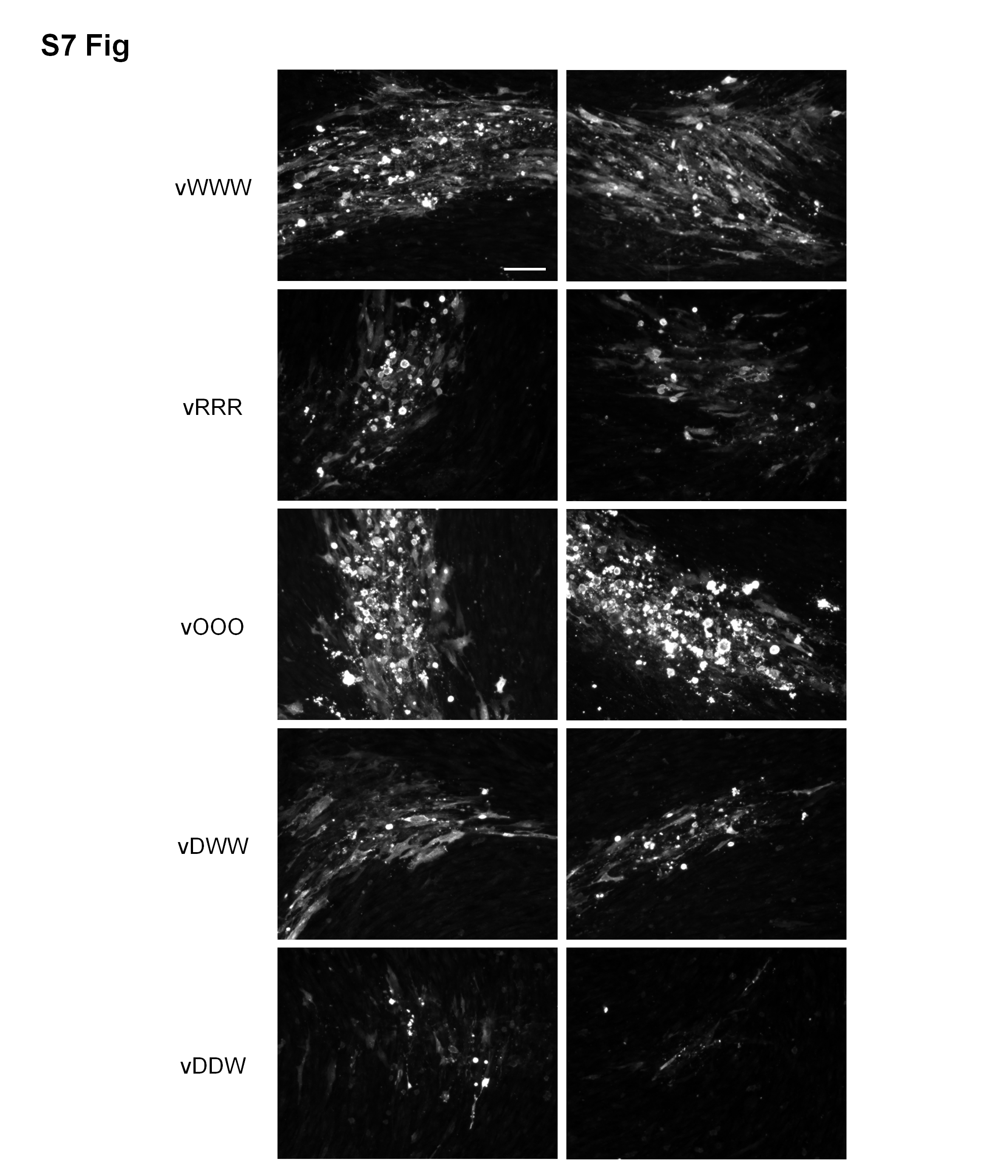

Supplement: S7 Fig — Plaques of the parental and mutant viruses in CEC 6 days post infection. Images were taken at 200-fold magnification. Scale bar, 100 μm. (TIF) [file ppat.1006857.s007.TIF]

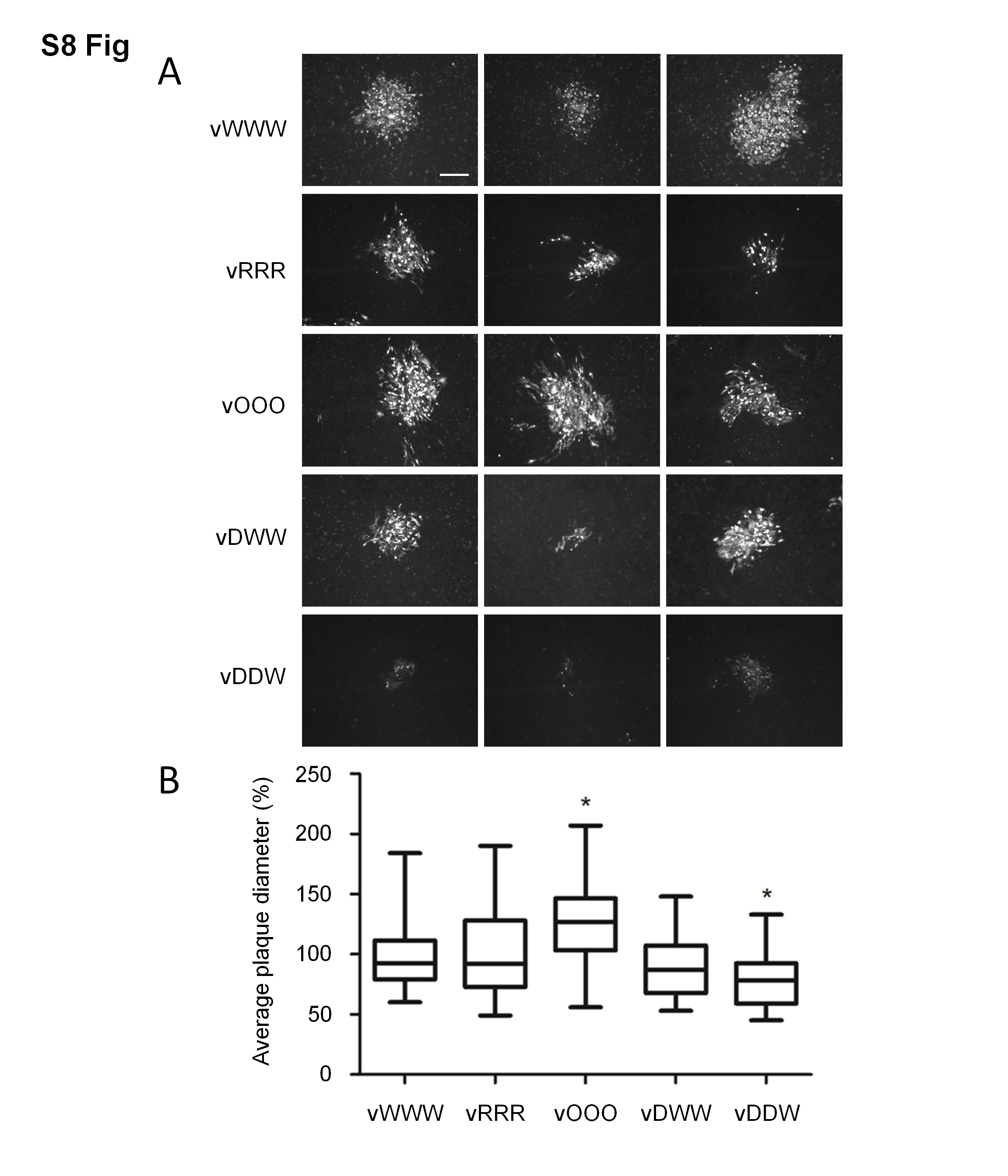

Supplement: S8 Fig — (A) Representative images of plaques formed by the parental and mutant viruses that were passaged 20 times serially at low multiplicity of infection in cell culture. Scale bar, 200 μm. (B) Serial passaging of viruses has not altered phenotype of mutant viruses. Similar to the viruses from early passages, plaque produced by the vOOO virus were larger, and plaques formed by the vDDW virus were smaller than those formed by the vWWW virus. The box-plot displays the distribution of relative plaque diameter normalized against the average plaque diameter of the parental virus. P-values were calculated using one-way ANOVA Bonferroni’s multiple comparison test, * indicates P<0.05. (TIF) [file ppat.1006857.s008.TIF]

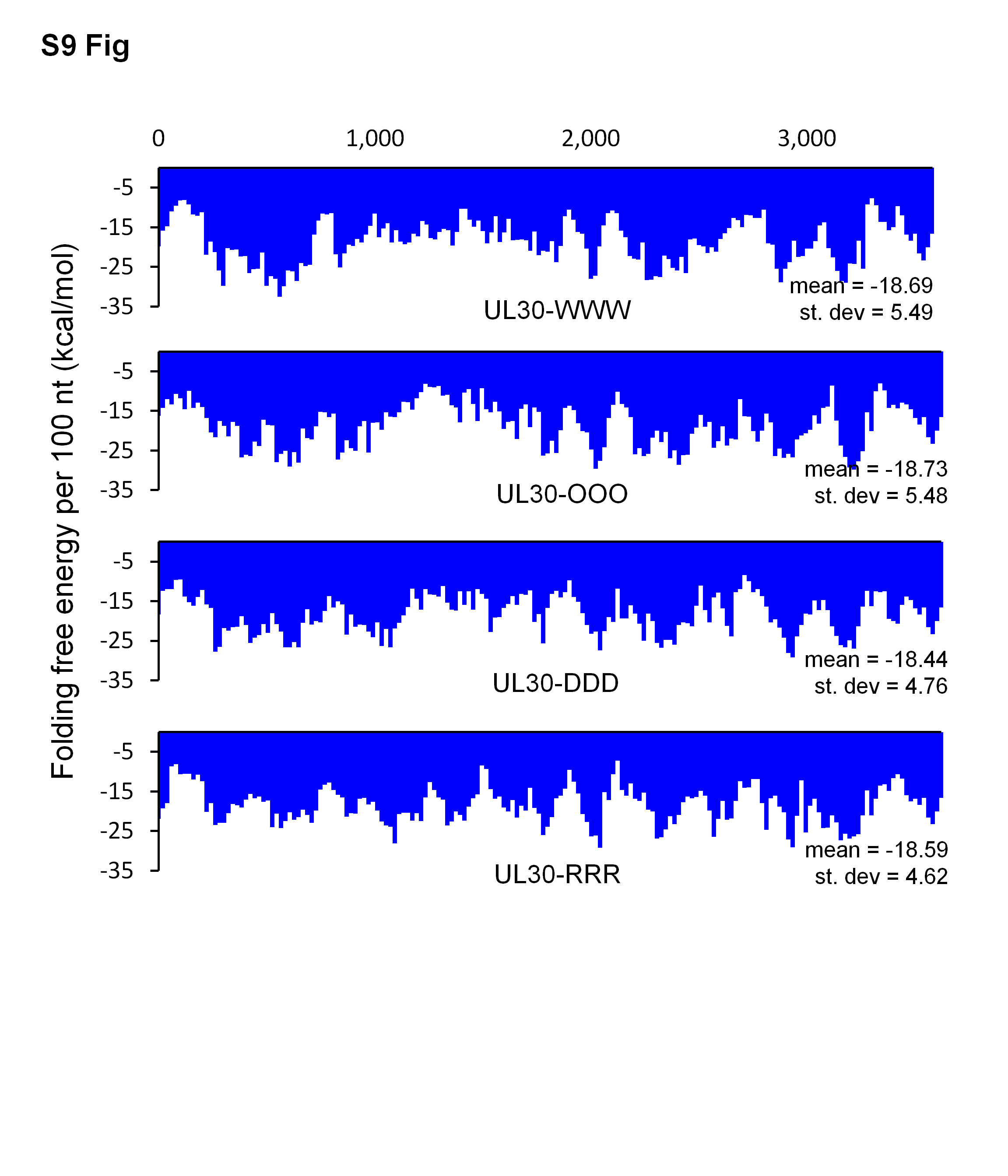

Supplement: S9 Fig — ΔG of the RNA encoded by the codon pair-optimized (OOO), -deoptimized (DDD) and -randomized (RRR) UL30 is similar to parental (WWW) UL30 gene. ΔG was calculated for 100 bp sequences that had a 80 bp overlap with each other. ΔG of any 100 bp fragment derived from the three recoded genes is not lower than -30 Kcal/mol. The parental and recoded sequences have similar mean ΔG: UL30-WWW = -18.7, UL30-OOO = -18.7, UL30-DDD = -18.4 and UL30-RRR = -18.6. (TIF) [file ppat.1006857.s009.TIF]
