# Supplementary material for: Attenuation of a very virulent Marek's disease herpesvirus (MDV) by codon pair bias deoptimization
Source: PLoS Pathog. 2018 Jan 29;14(1):e1006857. doi: 10.1371/journal.ppat.1006857 (PMC5805365; doi:10.1371/journal.ppat.1006857)
Supplement: S1 Table — (DOCX) [file ppat.1006857.s010.docx]

**S1 Table. Primers used in the study.**

| **Primer name** | **Sequence (5'-3')** | **Description** |
| --- | --- | --- |
| UL30-Start | CACGTGGGATAGTGTAGG | To sequence UL30 |
| UL30-End | TTAATATCGATGGGGAGTTGC | To sequence UL30 |
| UL30-seqF | ACACGGTACACTTCATCATGTGG | To sequence UL30 |
| UL30-seqR | TTGCTCATGTATGGCGTACTAAC | To sequence UL30 |
| W-seq-01 | GGTATCAGTATCGACTTTGC | To sequence UL30-WWW |
| W-seq-02 | GGAGCTGCTTCGCCCGATGG | To sequence UL30-WWW |
| W-seq-03 | AGTGTTAGAATTTGACAGCG | To sequence UL30-WWW |
| W-seq-04 | GTGATGGTCTTTGACTTTGC | To sequence UL30-WWW |
| W-seq-05 | AAGGGCTTAATTCTTTTGGC | To sequence UL30-WWW |
| D-seq-01 | ATGCGTAAAGTCGATACCGATAC | To sequence UL30-DDD |
| D-seq-02 | CGAAACAGCTTGAGCATGCAC | To sequence UL30-DDD |
| D-seq-03 | TCGCTAAGTTAGCCGGTATACTG | To sequence UL30-DDD |
| D-seq-04 | TCGTGTGTAATAGCGTATACGGG | To sequence UL30-DDD |
| D-seq-05 | GGGTACTCGCGGAAGCTTATAC | To sequence UL30-DDD |
| O-seq-01 | CGTGTTGCTGTCCATGTTTATG | To sequence UL30-OOO |
| O-seq-02 | AACACAAAGCAGCTAGAACATGC | To sequence UL30-OOO |
| O-seq-03 | CGTGTTTGCTTCGTTTGGC | To sequence UL30-OOO |
| O-seq-04 | CGAGATTATATTCACAAGCAGTGG | To sequence UL30-OOO |
| O-seq-05 | GTTCAGAACAACTTCCTGTGGTG | To sequence UL30-OOO |
| R-seq-01 | GGTGGCTGTTCATGTCTACGG | To sequence UL30-RRR |
| R-seq-02 | GCTCTTTTGTTCGCCTTGG | To sequence UL30-RRR |
| R-seq-03 | ATGCTTGCTGAGACTGGCGC | To sequence UL30-RRR |
| R-seq-04 | TGTTGTTGACTGTGCGAGATTAC | To sequence UL30-RRR |
| R-seq-05 | ACCTGTGGTTAAGGATCGAATAAG | To sequence UL30-RRR |
| Del UL30-Amp-F | GGGCCCAATTCTGACGATATCTGCATTGGCTACTATGTTCAAATATGTATCCGCTCATG | To replace UL30-WWW with Amp, forward primer |
| Del3/3 UL30-Amp-R | TGGAATAAATCTTCTCAACACAGTTTCCGTTATTTTCACGAACGAAAACTCACGTTAAGG | To replace UL30-WWW with Amp, reverse primer |
| Del2/3 UL30-Amp-R | CAACATATTGCGACCTATAGTAGTCACAGTCGCAGCGAACGAAAACTCACGTTAAGG | To replace first two thirds of UL30-WWW with Amp, reverse primer |
| Del1/3 UL30-Amp-R | ACTACTTTGAAACGTCTCTTTAAAGGTTTGTGGGAGGTCGAACGAAAACTCACGTTAAGG | To replace first third of UL30-WWW with Amp, reverse primer |
| Del2/3 UL30-Amp-F | AGTTGGAGCATGCATTATTGTTCGCTCTTGGTGCCTGTTTCAAATATGTATCCGCTCATG | To replace last two thirds of UL30-WWW with Amp, reverse primer |
| Del1/3 UL30-Amp-F | GTGGCGTATCGAATGGTTTATTACCTTGCATAGATGTCTTCAAATATGTATCCGCTCATG | To replace the last third of UL30-WWW with Amp, forward primer |
| UL30-WWW-BamHI | ATAGGATCCCACGTGGGATAGTGTAGG | To clone UL30-WWW into pUC57, forward primer |
| UL30-WWW-BamHI | TATGGATCCTACGTATCGTTGTGAACATTTCA | To clone UL30-WWW into pUC57, reverse primer |
| EP-UL30-WWW-F | ATATACTAGTACACGATGATACAAATTTAAGCAATTTACTAGGGATAACAGGGTAATCG | To insert AphAI into UL30WWW, forward primer |
| EP-UL30-WWW-R | ATATACTAGTGTCGTAAAGCATAAATTGTGTGCTTGCCAGTGTTACAACCAATTAACC | To insert AphAI into UL30WWW, reverse primer |
| EP-OOO-F | AGAGAATTTAAATACATTGCTCCCAAATGTTTGGATGATAAAGtagggataacagggtaatcgattt | To insert AphAI into pUC57-OOO, forward primer |
| EP-OOO-R | AGAGAATTTAAATTCATCAATGGTGGTGACATAAGTTATTGAAGCCAGTGTTACAACCAATTAACC | To insert AphAI into pUC57-OOO, reverse primer |
| EP-DDD-F | AGAGAACTAGTTGCGATATCGAAATTAATTGTACAGTCGATAAtagggataacagggtaatcgattt | To insert AphAI into pUC57-DDD, forward primer |
| EP-DDD-R | AGAGAACTAGTACAATGATGACATGGTGCGCGTACCTGCACTTGCCAGTGTTACAACCAATTAACC | To insert AphAI into pUC57-DDD, reverse primer |
| EP-RRR-F | AGAGAAGATCTCATGTCTTCTCTACAGCATAAATACCAAACAAtagggataacagggtaatcgattt | To insert AphAI into pUC57-RRR, forward primer |
| EP-RRR-R | AGAGAAGATCTGGATCACTACATCCTCCTCATTAGTAGCGCATGCCAGTGTTACAACCAATTAACC | To insert AphAI into pUC57-RRR, reverse primer |
| EP-DWW-F | TATATACGCGTATCATCCGATCTATTCCCTAAGCCTATTATAGGGATAACAGGGTAAT | To insert AphAI into pUC57-DDD, forward primer |
| EP-DWW-R | ATATAACGCGTTTAGCCATATCGTCGCCAGTCGTAACGAGCCAGTGTTACAACCAATTA | To insert AphAI into pUC57-DDD, reverse primer |
| EP-DDW-F | TATTATACTTAAGGATTGGTTAGCTATGCGTAAAGCGATTCGTAGGGATAACAGGGTAAT | To insert AphAI into pUC57-DDD, forward primer |
| EP-DDW-R | TATAATCTTAAGCAATATAGCGAGTAATGATTCGCGTATAGCCAGTGTTACAACCAATTA | To insert AphAI into pUC57-DDD, reverse primer |
| EP-WDD-F | TATATACCGGTATGATTACACTCGATATGTATAGTATATAGGGATAACAGGGTAATCG | To insert AphAI into pUC57-DDD, forward primer |
| EP-WDD-R | TATATACCGGTTGCCTTAAACTTACCTTTTTTTTGAGCCAGTGTTACAACCAATTAACC | To insert AphAI into pUC57-DDD, reverse primer |
| Ins UL30-F | ATAATAACTCTAAGTCACGTTTATGAAGTAGGA | For recombination cassette of UL30, forward primer |
| Ins UL30-R | CCGTGTAACTACAATCTTCTGTAAACG | For recombination cassette of UL30, reverse primer |
| Ins DWW-R | AAAATATTGTAACTACTACTTTGAAACGTCTCTTTAAAGGTTTGTGGGAGGTCGCATGCGCCTAATGCGAATA | For recombination cassette for first third of UL30 D, reverse primer |
| Ins DDW-R | TCACGTACTGTAAGCAACATATTGCGACCTATAGTAGTCACAGTCGCAGCTACGTCTATACATGGTAACAGTCCG | For recombination cassette for the first two thirds of UL30 D, reverse primer |
| Ins WDD-F | AGTTGGAGCATGCATTATTGTTCGCTCTTGGTGCCTGTGATTTACCCCAAACTTTTAAAG | For recombination cassette for last two thirds of UL30 D, forward primer |
| Ins WWD-F | TGTGGCGTATCGAATGGTTTATTACCTTGCATAGATGTCGCTGCGACAGTGACTACTATC | For recombination cassette for the last third of UL30 D, forward primer |
| UL30 forward | AAGCGGAATCGGTTTACAAG | To determine RNA levels of UL30 |
| UL30 reverse | GGAGTTGCTGTTAGAATACGGA | To determine RNA levels of UL30 |
| Probe UL30 | FAM-AACGGCCCTGTGATGCAGAA-TAM | qPCR Probe UL30 |
| UL29 forward | TGGGAGAAACGTGTGTTCAT | To determine RNA levels of UL29 |
| UL29 reverse | GCTGTTCTGCTACAAACCCA | To determine RNA levels of UL29 |
| Probe UL29 | FAM-CGCTATGTAATGACATGCCCTCGG-TAM | qPCR Probe UL29 |
| UL42 forward | TAACTATGGGCAGCGAACAC | To determine RNA levels of UL42 |
| UL42 reverse | TTAATGGGTTCTTCACGCAA | To determine RNA levels of UL42 |
| Probe UL42 | FAM-CAACGTTCCCAACAAACGATCCTG-TAMRA | qPCR Probe UL42 |
| BFP forward | AACGGCCCTGTGATGCAGAA | To determine RNA levels of BFP |
| BFP reverse | CGAGCTTCAGGGCCATGTC | To determine RNA levels of BFP |
| ICP4 forward | CGTGTTTTCCGGCATGTG | To determine MDV genome copies in chicken whole blood |
| ICP4 reverse | TCCCATACCAATCCTCATCCA | To determine MDV genome copies in chicken whole blood |
| Probe ICP4 | FAM-CCCCCACCAGGTGCAGGCA-TAM | qPCR Probe ICP4 |
| iNOS forward | GAGTGGTTTAAGGAGTTGGATCTGA | To determine iNOS genome copies in chicken whole blood |
| iNOS reverse | TTCCAGACCTCCCACCTCAA | To determine iNOS genome copies in chicken whole blood |
| Probe iNOS | FAM-CTCTGCCTGCTGTTGCCAACATGC-TAM | qPCR Probe iNOS |
