# Supplementary material for: Attenuation of a very virulent Marek's disease herpesvirus (MDV) by codon pair bias deoptimization
Source: PLoS Pathog. 2018 Jan 29;14(1):e1006857. doi: 10.1371/journal.ppat.1006857 (PMC5805365; doi:10.1371/journal.ppat.1006857)
Supplement: S1 Appendix — The coding sequence of the parental UL30 gene was divided into three equally long segments (1,221 bp), and each segment was recoded independently. The second 1,221 bp UL30 segment is in lowercase. The last 201 bp of the coding sequence that were not subjected to recoding are underlined. (DOCX) [file ppat.1006857.s011.docx]

**S1 Appendix**

Sequences of the recoded UL30 genes. The coding sequence of the parental UL30 gene was divided into three equally long segments (1,221 bp) and each segment was recoded independently. The second 1,221 bp segment is in lowercase. The last 201 bp of the coding sequence that were not subjected to recoding are underlined.

**>UL30-RRR**

ATGTCTGTTGATGGCACGAAAACGTTTTTTAATCCGTATATCGGTGCCCGTAAGAGGAGTCTTGAAGCACGAAATGGATTATCCTTCTCTACTGGACAGAACTATGACGAAAAAAACAATCGCCGTGACCGAAATTCAATAACTTATGTCACCACTATAGATGAA*TTTAAA*TATATTGCCCCGAAATGTTTAGATGATAAAGATGTAAAACAAAAAGGTACTCATATAGGAAAACTAAAGCGAAGCCCAGTACTTTATAAAAACGGGGAGGAATATGTATTCCTGAATTTTGAAGATTGTGAGGACGTATGGCCCCGCCGATGCTCTATTTGGAATAACCGGAGCTTCTTGCCGGCAGATTTTGATCCGAGATTTTCACGTTTTCATGTGTATGACATGATTGAAACCGTAGAATTTGCATCAGCAGCAATTGATCGTGATAAAAACAGGTTTCTCGAACTTTTACGGCCTATGGGAACAATCGTCACAATGATGGGAATCACTGAATGCGGCAAAAGGGTGGCTGTTCATGTCTACGGAATTAAACCTTATTTTTACATGCGTAAAGTAGATACTGATACAATCTGTGGATCTCGATGTCCGAGAGAACTAGCGGAAAAATTGGCCAACGTGGTAAGGTCGTCAGTAAACGAAGTTGCAAACGCAAAACGTTTTTGTACCCCCGTAACTAGGACAGTTTCTGCAGACTGTTTCGAAGTCGACGTTGTACAGAGAAAAGATATTTATTATTATGGCACAGGCCATGACGAATTTTATCGAGTAAAGTCCCAGTCAGGC**AAATTT**ATAACTCTACTATGTGATAATTTTTACCCATCCATTATAAAATATGAAGGCAATATTGATGCTATCACTCGCATGGTACTGGATAATAATGGATTCTCCACCTTTGGCTGGTATTCTTTCAAGGTGGGAAACAATGGAGAAAAAGTACAAGTACGGGCACCCTGTCATCATTGTACGTCATGCGATATCGAGATTAACTGCACTGTCGATAATCTAATTGGTTACCCCGAAGATGATGCATGGCCAGACTATAAACTACTTTGCTTTGACATAGAGTGCAAGAGTGGAGGAGTCAACGAATGCGCCTTTCCATGCGCTACTAATGAGGAGGATGTAGTGATCCAGATCTCATGTCTTCTCTACAGCATAAATACCAAACAATTGGAGCATGCTCTTTTGTTCGCCTTGGGAGCATGTgatttgccccaaacttttaaggaaacttttcaaagctcgtataatattttacccatagttctggaatttgatagcgaatttgagttgctcttggcatttatgactttcattaaacaatatgcaccagaatttgttacgggttacaatatagt**AAATTT**cgactgggcatttattgtaaccaaattaacaacggtgtataatatgcggttggatggttatggtgtcgtaaaccaaaagggaatg*TTTAAA*gtatgggatgccggcaccaatcgatttcagaagaaaggg**AAATTT*AAA***gcaactggtatgataacgttagatatgtattctatagcgacagagaaattaaaattacaaagctataaattagacgtggtagccgaagccgcgctcggagaacggaagaaagaactttcctacaaagaaataccgagtcactttgcagctggccctgagaagagggggattataggcgaatattgtcttcaagattccttattggtcggtaaactgttt*TTTAAA*tacattccacatttagaattaagtgcaatagccaaattggcgggaatattattatcaaaggctatttttgatggccaacagatacgtgtgtatacatgcttgctgagactggcgcgcagtcacggatttatattaccagaaaaaaataaa**AAATTT**gcagaaactgtcagtctaacatgtgaggaagaccaaaccgaaatctgtgaacacgattcgccccaagagcccatacataatattaagcaaagcagcttgtgccattcaaattccggcagaactattggataccagggcgccaaggtcctcgatccaatatcaggctttcatgtggaccctgtgatggtgtttgattttgcaagcctatatccctcaatcattcaagctcataatttatgttttacgacattagtacatgatgatac**AAATTT**gtctaatctacgccctcaagacgattatttggaaattaatgtgcaaggaaagcttcttcgtttcgttaaaccccacattagagaatcgttactggccatattgttgaaagactggttagccatgcgtaaagcaattcgcgctaagatacccgaaagttgcgatgaaatcgctgttctgttagataaacagcaggcagcgattaaagtagtctgtaacagtgtgtatggattttgtggagtgtcaaatggacttttaccgtgcattgatgtgGCTGCTACTGTAACTACAATAGGGCGAAACATGTTGTTGACTGTGCGAGATTACATACATAAACAATGGGGAACCCGAGACGCACTGCTAAGGGAATTTCCTAATCTTTCTAATTTTATGCGGCCTGAAGACTATTCCGTATCCGTGATTTATGGAGATACGGATTCAGTATTTATAAAG*TTTAAA*GGGGTGGATATACATGGTCTTGTAACTACAGGAGACGACATGGCCAAGCGTGTTTCTTCAGATTTGTTTCCGAAACCAATTAAGCTCGAGTGTGAAAAAACTTTCAACAAGTTATTACTCATAACTAAAAAAAAATATATGGGAACAATACACGGGGGAAGAATGTTGATGAAAGGCGTAGACATTGTACGCAAAAATAATTGCCGTTTCATTAACACATATGCGAAAAAGCTCTCTGATCTCTTATTTTTGGATGATACAGTGGCCAAAGCTGCCGCGACTGTCGCAGAAAAACCACCAAGTTTCTGGGCCACCTCACCTCTTCCTGAAGGATTAAACAGCTTTGGTGGTGTTCTGGCAGAAGCTTACACTCGGATGATGATAAATAATATTACAGAAGTCGAAGATTTTGCTATGTCCGCCGAGCTCTCGCGCCCCCCGGATGCTTATACCAATAAGCGCATTCCTCATTTGACTGTATACTATAAACTGGCAATGCGGTCAGAACAGCTACCTGTGGTTAAGGATCGAATAAGTTATGTTATTGCGGCTGCGACACCCGAAGTCGTAAGAGACTCCGCTAGAGTGGCTGAATTCAGGGGGGAGTTGGACTTGTGCCATCAAAATTCCAACACTTCATGCCCCGGCGACTCTGTAATGACTAACAAAGAAACATATGTTAGACATTCTCCGAGAAATAAGTTGCTGATCAGTGATATGGCGGAAGATCCTAAATATTTGTTAGCCAATAACATACCCCTTAATACGGATTATTATTTGAGCCATCTACTAGGAACTCTTTGTGTGACT*TTTAAA*GCACTTTTCGGGAATGATGTGAAAATAACGGAAACTGTGTTGAGAAGATTTATTCCAGAAACGTTTACAGAAGATTGTAGTTACACGGAACGTGTCTCCAGTGAAATGTTCACAACGATACGAAGCGGAATCGGTTTACAAGTCAACGAGGAGGAAGAAACTCGTCGAAAGCTGAATATAGCTTTCCGTATTCTAACAGCAACTCCCCATCGATATTAA

**>UL30-OOO**

ATGTCTGTAGATGGCACAAAAACTTTCTTCAACCCCTACATTGGAGCAAGGAAACGTTCTTTGGAAGCACGAAATGGTTTATCATTTTCAACTGGACAAAATTATGATGAGAAGAACAACCGGCGGGACAGAAATTCAATAACTTATGTCACCACCATTGATGAA*TTTAAA*TACATTGCTCCCAAATGTTTGGATGATAAAGATGTAAAACAAAAAGGAACTCATATTGGAAAACTAAAACGTTCTCCAGTACTTTATAAAAATGGAGAAGAATATGTGTT*TTT****AAATTT***TGAAGACTGTGAAGATGTCTGGCCGCGCCGCTGCTCCATCTGGAACAACAGAAGTTTTCTTCCAGCAGATTTTGATCCTCGTTTTTCTCGATTTCATGTTTATGACATGATAGAAACTGTAGAATTTGCATCAGCAGCAATAGATCGTGACAAGAACAGATTTCTAGAGCTGCTGCGGCCGATGGGCACCATTGTAACGATGATGGGAATAACAGAATGTGGGAAGCGTGTTGCTGTCCATGTTTATGGAATAAAACCATATTTTTATATGAGGAAAGTAGATACTGATACCATCTGTGGCAGCCGATGTCCTCGAGAGCTAGCAGAGAAGCTAGCAAATGTGGTGCGCTCCTCTGTAAATGAAGTAGCAAATGCAAAACGTTTCTGCACACCAGTGACGAGGACTGTCTCGGCGGACTGCTTTGAGGTCGACGTAGTACAGAGGAAAGATATTTATTATTATGGCACTGGACATGATGAATTTTATCGAGTAAAATCTCAGAGTGGA**AAATTT**ATAACTTTGCTTTGTGACAACTTCTACCCCAGCATCATCAAATATGAAGGAAATATTGATGCCATCACCAGGATGGTTTTGGACAACAATGGCTTCTCCACGTTTGGCTGGTACAGCTTCAAAGTGGGCAACAATGGAGAAAAAGTACAAGTACGAGCCCCGTGCCATCATTGTACTTCATGTGATATTGAGATCAACTGCACTGTAGATAATTTAATTGGTTATCCGGAAGATGATGCCTGGCCGGACTACAAACTACTTTGTTTTGATATTGAGTGCAAATCAGGAGGAGTAAATGAATGTGCCTTCCCCTGTGCAACAAATGAAGAAGATGTTGTCATCCAGATCTCCTGCCTCCTCTATTCAATAAACACAAAGCAGCTAGAACATGCTTTGCTTTTTGCTCTTGGTGCCTGCgacttgccacaaact*TTTAAA*gaaacttttcagagctcctacaatattttaccaatagtattagaatttgacagtgaatttgaattacttctagcctttatgacgtttataaaacaatatgcgccggagtttgtaacaggatataatattgt**AAATTT**tgactgggcatttattgtcaccaaattaacaactgtttataatatgagattagatggttatggtgtggtgaatcaaaaaggaatg*TTTAAA*gtatgggatgctggaacaaatcgttttcagaagaaagga**AAATTT*AAA***gccacgggcatgataactttggacatgtacagcattgcaacagaaaaattaaaactacaaagttataaactagatgtggtggcggaggcggcgctgggagaaaggaagaaagaattatcatataaagaaatacccagccactttgctgctggaccagaaaagcggggcattattggagaatactgtttgcaagattctttgcttgttggaaaattattt*TTTAAA*tatattcctcatttagaattatcagcaatagcaaagctggcgggcattttattgtcaaaagcaatatttgatgggcagcaaatacgtgtctatacgtgtttgcttcgtttggcaagatcccatggcttcattttaccagagaagaataagaagtttgcagagacggtgtccctcacctgtgaagaagatcaaacagaaatatgtgaacatgattcaccccaggagcccattcataatataaaacaaagttcgctgtgccacagcaacagtggccgcaccattggttatcaaggagcaaaagtattggatcccatctcgggctttcatgtggatcctgtgatggtgtttgattttgcctcgctctaccccagcataatacaagctcacaacctctgcttcactactttggtacatgatgacac**AAATTT**atc**AAATTT**acggccgcaggatgattatttagaaataaatgtacaagggaagcttcttcgatttgtgaagccccacatcagagaaagtttgctggccattttattaaaagactggctggccatgcgcaaagccatccgcgccaagattcctgaaagttgtgatgaaatagctgttttgttagataaacaacaagcagcaataaaagttgtctgcaattctgtctatggcttctgtggtgtcagcaatggtttgctgccctgcattgatgtgGCGGCGACTGTAACAACTATTGGCCGCAACATGTTGCTAACTGTACGAGATTATATTCACAAGCAGTGGGGAACAAGAGATGCTTTGCTAAGAGAATTTCCAAATCTCTCCAACTTCATGCGGCCCGAGGATTATTCTGTTTCTGTAATATATGGGGACACTGATTCTGTCTTCATA**AAATTT*AAA***GGAGTAGATATTCATGGACTGGTGACAACTGGAGATGACATGGCAAAACGTGTTTCTTCAGATCTCTTCCCCAAGCCCATAAAACTAGAATGTGAAAAAACTTTTAATAAACTTCTTTTAATAACAAAGAAGAAATACATGGGGACTATTCATGGTGGCCGCATGCTGATGAAAGGAGTTGATATTGTGAGGAAGAACAACTGCCGCTTCATAAATACTTATGCCAAGAAACTTTCAGATTTGTTGTTTTTGGATGACACGGTGGCAAAAGCTGCTGCTACTGTAGCAGAAAAACCTCCTTCCTTTTGGGCTACTTCTCCTTTACCTGAAGGATTAAATAGTTTTGGTGGTGTTTTGGCTGAAGCCTACACCAGGATGATGATAAATAATATAACAGAAGTAGAAGATTTTGCCATGTCGGCGGAGCTCTCCCGGCCGCCGGATGCCTACACCAACAAAAGAATACCTCATTTAACTGTTTATTATAAACTTGCCATGCGTTCAGAACAACTTCCTGTGGTGAAGGACAGAATATCCTATGTAATTGCTGCGGCGACGCCAGAAGTGGTGCGGGACAGTGCTCGAGTAGCAGAATTTCGAGGGGAGCTGGACCTCTGCCATCAAAACAGCAACACCAGCTGCCCGGGGGACAGTGTCATGACAAATAAAGAAACATATGTACGACATTCACCAAGAAATAAGCTGCTCATCTCCGACATGGCAGAAGATCCCAAATATTTGCTAGCCAATAATATTCCTTTGAATACAGATTATTATTTATCACATTTGCTTGGAACTTTGTGTGTCACT*TTTAAA*GCTTTGTTCGGGAATGATGTGAAAATAACGGAAACTGTGTTGAGAAGATTTATTCCAGAAACGTTTACAGAAGATTGTAGTTACACGGAACGTGTCTCCAGTGAAATGTTCACAACGATACGAAGCGGAATCGGTTTACAAGTCAACGAGGAGGAAGAAACTCGTCGAAAGCTGAATATAGCTTTCCGTATTCTAACAGCAACTCCCCATCGATATTAA

**>UL30-DDD**

ATGTCCGTAGACGGAACTAAGACTTTTTTTAATCCGTATATAGGCGCACGTAAACGATCACTCGAGGCACGTAACGGACTATCTTTTTCTACAGGCCAAAATTACGACGAAAAAAATAATAGACGCGATCGTAACTCAATTACTTATGTGACTACTATCGATGAG*TTTAAA*TATATAGCGCCAAAATGTCTTGACGATAAAGACGTAAAACAGAAAGGCACACATATCGGTAAGCTTAAACGATCACCCGTACTATATAAAAACGGCGAAGAGTACGTTTTTCTTAATTTCGAAGATTGCGAAGACGTATGGCCTAGACGATGTTCAATTTGGAATAATCGGTCTTTTCTGCCAGCCGATTTCGATCCGCGATTTTCTAGGTTTCATGTATACGATATGATCGAAACCGTCGAATTCGCTAGCGCCGCAATCGATCGCGATAAAAATAGGTTTCTCGAACTATTGCGACCTATGGGGACTATCGTAACTATGATGGGAATTACCGAATGCGGAAAACGGGTAGCCGTACATGTATACGGAATTAAACCGTATTTTTATATGCGTAAAGTCGATACCGATACTATATGCGGATCTAGGTGTCCGAGAGAGCTTGCCGAAAAATTGGCTAACGTAGTTAGGTCTAGCGTTAACGAAGTCGCTAACGCTAAACGGTTTTGTACTCCCGTTACGCGAACCGTATCCGCCGATTGTTTCGAAGTCGACGTAGTGCAACGTAAAGATATATATTATTACGGAACCGGACATGACGAATTTTATAGGGTTAAGTCACAATCCGGTAAGTTTATTACATTGTTATGCGATAATTTTTATCCGTCAATTATAAAATATGAGGGTAATATAGACGCAATTACGCGTATGGTGCTTGATAATAACGGATTTTCGACATTCGGATGGTATAGC*TTTAAA*GTCGGAAATAACGGCGAAAAAGTGCAGGTACGCGCACCATGTCATCATTGTACTAGTTGCGATATCGAAATTAATTGTACAGTCGATAACCTAATCGGATATCCCGAAGATGACGCATGGCCCGATTATAAATTGTTGTGTTTCGATATCGAATGTAAGTCCGGAGGCGTAAACGAATGCGCATTTCCATGCGCAACTAACGAAGAGGATGTAGTGATACAGATTAGTTGTCTGTTATATTCAATTAATACGAAACAGCTTGAGCATGCACTACTATTCGCATTAGGCGCATGCgatttaccccaaact*TTTAAA*gagacatttcaatcctcttataatatattgcctatagtgttagagtttgatagcgaattcgaattgttgttagcgtttatgacttttattaaacaatacgcacccgaatttgtgactgggtataatatagtgaattttgattgggcatttatcgttacgaaattgactacggtatataatatgcgattagacggatacggagtcgttaatcaaaagggtatg*TTTAAA*gtgtgggacgcaggcactaatcggtttcaaaaaaaaggtaagtttaaggcaaccggtatgattacactcgatatgtatagtatagcgaccgaaaaattgaaattgcaatcttataaactcgatgtggtcgccgaagccgcattaggcgaacgtaaaaaagaattgagttataaggaaattccgtcacattttgccgcaggacccgaaaaaagaggcattataggcgaatattgtttacaggatagtctgttagtcggaaaattgttt*TTTAAA*tatataccacaccttgagttatccgcaatcgctaagttagccggtatactgttatcgaaagcgatttttgatggccaacagatacgcgtatatacatgtctattgcggttagctaggtcacacggatttatattacccgaaaaaaataaa**AAATTT**gccgaaacagtgagtctgacatgcgaagaggatcaaaccg**AAATTT**gcgaacacgatagcccacaggaacctatacataatattaaacaatcgtcattgtgtcatagtaattccggacgtacaatcggatatcagggcgcaaaagtgttagatccgattagcggatttcatgtcgatcccgttatggtgtttgatttcgctagcctataccctagcattatacaagcgcataatctatgttttacgactctggtacacgacgatacgaatttgtcgaatcttagaccccaagacgattatcttgagattaacgtgcaaggtaagttacttagattcgttaagccacatatacgcgaatcattactcgctatattgcttaaggattggttagctatgcgtaaagcgattcgcgcaaaaattcccgaatcatgcgacgaaattgccgtactgttagataagcaacaggccgcaattaaggtcgtgtgtaatagcgtatacgggttttgcggcgtaagtaacggactgttaccatgtatagacgtaGCTGCGACAGTGACTACTATCGGACGAAATATGTTATTGACCGTACGCGATTATATACATAAACAATGGGGTACTAGAGACGCATTGTTGCGCGAATTTCCGAATCTGTCTAACTTTATGCGACCTGAGGATTATAGCGTATCAGTGATATACGGAGATACCGATAGCGTATTTATTAAGTTTAAGGGGGTAGACATACATGGACTCGTTACGACTGGCGACGATATGGCTAAACGCGTATCATCCGATCTATTCCCTAAGCCTATTAAACTCGAATGCGAAAAGACATTTAATAAATTGTTGTTGATTACTAAAAAAAAATATATGGGTACTATACACGGAGGGCGTATGCTTATGAAGGGAGTCGATATAGTGCGAAAAAACAATTGTCGGTTTATTAATACTTATGCGAAAAAATTGTCCGATCTGTTATTCTTAGACGATACAGTCGCTAAGGCCGCAGCTACCGTAGCGGAAAAACCTCCTAGTTTTTGGGCTACATCTCCATTGCCCGAAGGGCTTAACTCATTCGGAGGGGTACTCGCGGAAGCTTATACTAGAATGATGATTAACAATATTACTGAGGTCGAAGATTTCGCAATGTCTGCCGAATTGTCTAGACCACCTGACGCATATACTAATAAACGGATTCCGCATCTTACAGTGTATTATAAACTCGCTATGCGATCCGAACAATTGCCCGTAGTGAAAGATCGGATATCATACGTGATAGCCGCAGCGACACCCGAAGTGGTTAGGGATTCCGCTAGGGTAGCCGAATTTAGAGGCGAACTCGATCTATGCCATCAGAATTCGAATACGTCTTGTCCCGGAGACTCAGTTATGACTAACAAAGAGACATACGTTAGACATAGTCCACGTAATAAATTGCTTATATCCGATATGGCCGAAGACCCTAAGTATCTGTTAGCCAATAACATACCGCTTAATACTGACTATTATCTTAGTCATCTGTTAGGGACTCTATGCGTTACATTTAAGGCTCTATTCGGTAACGATGTGAAAATAACGGAAACTGTGTTGAGAAGATTTATTCCAGAAACGTTTACAGAAGATTGTAGTTACACGGAACGTGTCTCCAGTGAAATGTTCACAACGATACGAAGCGGAATCGGTTTACAAGTCAACGAGGAGGAAGAAACTCGTCGAAAGCTGAATATAGCTTTCCGTATTCTAACAGCAACTCCCCATCGATATTAA

| **Gene** | **AAATTT** | **TTTAAA** | **TTTAAA + AAATTT** |
| --- | --- | --- | --- |
| RRR | 5 | 6 | 11 |
| OOO | 7 | 8 | 15 |
| DDD | 2 | 5 | 7 |
